# Single-dose DSS-induced inflammation enhances colorectal tumorigenesis in *APC* and *KRAS* mutant mice

**DOI:** 10.1038/s41598-025-25577-1

**Published:** 2025-11-24

**Authors:** Kazuki Ishibashi, Yuji Urabe, Takahiro Uda, Yukiko Sako, Tomoyuki Gurita, Satoshi Masuda, Yoshiki Hatsushika, Takeo Nakamura, Hirona Konishi, Akiyoshi Tsuboi, Hidenori Tanaka, Ken Yamashita, Yoshihiro Kishida, Yuichi Hiyama, Hidehiko Takigawa, Toshio Kuwai, Hiroaki Niitsu, Takao Hinoi, Shiro Oka

**Affiliations:** 1 Department of Gastroenterology, Graduate School of Biomedical and Health Sciences, Hiroshima University Hospital, Hiroshima University, 1-2-3, Kasumi, Minamiku, Hiroshima, 734-8551 Japan; 2 Department of Gastrointestinal Endoscopy and Medicine, Hiroshima University Hospital, Hiroshima, Japan; 3 Department of Clinical and Molecular Genetics, Hiroshima University Hospital, Hiroshima, Japan

**Keywords:** Apc, Colorectal cancer, DSS, Inflammation, Kras, Cancer, Genetics, Gastroenterology

## Abstract

Colorectal cancer (CRC) arises through interactions between driver mutations, such as in *APC* and *KRAS*, and the tumor microenvironment (TME), including inflammatory factors. While chronic inflammation is a known risk factor, the role of transient mild inflammation in tumorigenesis remains unclear. This study assessed the impact of mild inflammation on CRC development using genetically modified *KRAS* mutant (mut), *APC* mut, and *APC; KRAS* double mut mouse models. Mice received tamoxifen at six weeks and were evaluated with or without a 5-day administration of 1.5% dextran sulfate sodium (DSS). Mice were sacrificed at 20 weeks, and tumor number, size, location, histology, immunofluorescence, and RNA sequencing were analyzed. Tumors were absent in *APC* and *KRAS* mut mice not treated with DSS, while *APC; KRAS* mut mice developed small proximal colon tumors. DSS-treated *KRAS* mut mice remained tumor-free, but *APC* and *APC; KRAS* mut mice developed multiple tumors throughout the colon. In *APC; KRAS* mut mice, DSS significantly increased tumor number and size in the proximal colon. Although DSS did not alter immune infiltration in proximal tumors, regulatory T cells and M2 macrophages were elevated in *APC; KRAS* compared to *APC* mutants, suggesting immunosuppressive TME. These findings indicate that transient inflammation promotes CRC development in *APC* mutant mice.

**Supplementary Information:**

The online version contains supplementary material available at 10.1038/s41598-025-25577-1.

## Introduction

Colorectal cancer (CRC) is one of the most prevalent malignancies worldwide and arises from a multistep process involving the accumulation of genetic alterations and environmental influences^1,2^. Among various oncogenic pathways, the adenoma–carcinoma sequence is the most widely recognized, with mutations in the *Adenomatous Polyposis Coli* (*APC*) gene initiating tumorigenesis, followed by mutations in *Kirsten rat sarcoma viral oncogene homolog* (*KRAS*), *tumor protein (TP53)*, and other driver genes^3–5^. Although such genetic alterations are essential for tumor initiation, accumulating evidence suggests that they are insufficient on their own to induce tumorigenesis, highlighting the importance of tumor microenvironmental factors, including inflammation, in colorectal carcinogenesis^5^.

Recent studies have elucidated the multifaceted mechanisms by which inflammation contributes to tumor initiation, progression, and metastasis^6,7^. Chronic inflammation, such as that observed in ulcerative colitis, is well known to promote CRC development^8^. However, it remains unclear whether even mild or transient inflammation can also enhance tumorigenesis. Investigating such subtle effects requires experimental systems that allow precise dissection of genetic and environmental interactions.

While recent advances in sequencing and modeling are increasingly revealing the complex and diverse nature of colorectal tumorigenesis^9–12^, it remains challenging to dissect how specific environmental stimuli, such as inflammation, modulate tumor initiation and progression in the presence of defined driver mutations. In this context, genetically engineered mouse models (GEMMs) are essential tools to evaluate the interactions between genetic alterations and external factors under physiologically relevant conditions^13,14^. The *Apc*^*min/+*^ mouse model, one of the earliest GEMMs, has been instrumental in elucidating intestinal tumorigenesis^15,16^. However, its limitations include the predominance of small intestinal tumors and off-target effects in hematopoietic and other tissues^17^, which do not faithfully mimic human familial adenomatous polyposis (FAP), characterized by colorectal adenomas and carcinomas^18^.

To overcome these limitations, we have developed GEMMs for colonic neoplasia by targeting genetic recombination, specifically in the colonic epithelial cells. To that end, we first developed the *CDX2P 9.5-NLS-Cre* (CPC) mouse harboring a *Cre* transgene fused to the 9.5 kb *CDX2* promoter region (*CDX2P-9.5)*, which exhibits colonic epithelial cell-specific transcriptional activity^17^. By crossing CPC with *Apc flox* mice, *CDX2P 9.5-NLS-Cre; Apc*^*flox/+*^ (*CPC; Apc*) mice develop multiple colonic neoplasm at the distal colon and cecum through loss of heterozygosity of the wild-type *Apc* allele after birth. However, combining other floxed alleles—such as mutant *Kras*,* Braf*, and *Tgfbr2 —*with CPC; APC mice resulted in fetal lethality due to *CDX2P-9.5’*s transcriptional activity, which initiates from embryonic development^19,20^. To address this, we recently developed a *CDX2P 9.5-CreER*^*T2*^ mouse in which genetic recombination in the colonic epithelium is induced by tamoxifen treatment. This model enables both spatially and temporally controlled gene recombination in the large intestine, offering a more accurate representation of human colorectal tumorigenesis compared to models like *Villin-CreER*^*T2*^ or *Lgr5-CreER*^*T2*^.

Building on this platform, the present study investigates whether short-term, low-level inflammation, induced by a 5-day administration of dextran sulfate sodium (DSS), can promote tumorigenesis in mice harboring *APC* and/or *KRAS* mutations.

In this study, we aimed to clarify whether mild, transient inflammatory stimuli—without prolonged colitis—could cooperate with *APC* and *KRAS* mutations to promote colorectal tumor formation.

## Materials and methods

### Ethics statement

This study was conducted in strict accordance with the Guide for the Care and Use of Laboratory Animals, and the Committee of Hiroshima University.

All protocols were approved by the Institutional Animal Care and Use Committee of Hiroshima University (Permit Number: A24-33). These approvals include the planned experimental design, statistical considerations, and procedures (including schedules of humane killing, in this case using intraperitoneal administration of a medetomidine-midazolam-butorphanol mixture at doses of 0.5 mg/kg medetomidine, 4.0 mg/kg midazolam, and 5.0 mg/kg butorphanol, prior to neck dislocation), as well as the intended outcomes. All procedures were conducted in accordance with institutional, national, and international guidelines and regulations, and are reported in full accordance with the ARRIVE guidelines (https://arriveguidelines.org).

### Experimental animals

All the mice were housed under specific pathogen-free conditions. They were fed Teklad Mouse Breeder Diet 8626, with autoclaved water supplied ad libitum. The breeding room was maintained at a constant temperature of 23 °C ± 2 °C, relative humidity of 50 ± 5%, 15–20 air changes per hour, and a 12 h light/dark cycle, with lights on at 8:00 am. Four to five mice were housed per cage with chopped-wood bedding^21^.

We utilized *CDX2P9.5-CreER*^*T2*^ mice as described previously^22^. These Cre mice were crossed with *Apc* ^*flox/flox*^ mice^23^ and *Kras* ^*LSL−G12D/+*^ mice (C57BL/6J)^24^to obtain *CDX2P9.5-CreER*^*T2*^;*Apc*^*flox/+*^;*Kras*^*LSL−G12D/+*^ (*APC; KRAS* mut), *CDX2P9.5-CreER*^*T2*^;*Apc*^*flox/+*^ (*APC* mut), and *CDX2P9.5-CreER*^*T2*^;*Kras*^*LSL−G12D/+*^ (*KRAS* mut) mice^25^. To minimize sex-related variability, only male mice were used in all experiments. All mouse strains used in this study were backcrossed for more than 10 generations onto the C57BL/6J background and were originally derived from C57BL/6J mice.

### Cre expression and DSS-induced inflammation analysis

Separate experiments were conducted to evaluate the distribution of Cre expression and the extent of DSS-induced inflammation.

Tamoxifen (Sigma-Aldrich) was dissolved in 10 mg/mL corn oil, and single doses of 15 mg/kg tamoxifen were administered intraperitoneally to *CDX2P9.5-CreERT2* transgenic mice at the age of 6 weeks.

Three days following tamoxifen administration, the mice were sacrificed, and their colons were collected. Samples were fixed in 4% paraformaldehyde (PFA) and embedded in paraffin. For immunofluorescence staining, the paraffin slides were deparaffinized and subjected to antigen retrieval using citrate buffer (pH 6). Anti-Cre antibody (Cell Signaling, D7L7L, 1:100) was then applied overnight at 4 °C, followed by incubation with a fluorescent secondary antibody (AF568, Invitrogen, A-11011, 1:250) for 1 h. The slides were counterstained with 4′,6-diamidino-2-phenylindole (DAPI), mounted with an antifade medium.

To assess the distribution of DSS-induced colonic inflammation, wild-type C57BL/6J mice were administered 1.5% DSS in their drinking water for 5 days. Mice were sacrificed 7 or 28 days after completion of DSS administration. Colonic tissues were collected, fixed in 4% PFA, embedded in paraffin, and sectioned for hematoxylin and eosin (H&E) staining. The extent of inflammation was determined through histological examination.

### Tamoxifen treatment, induction of colitis in mice, and general assessment of colitis and tumorigenesis

Single doses of 15 mg/kg tamoxifen were intraperitoneally injected into mice carrying the *CDX2P9.5-CreER*^*T2*^ transgene at 6 weeks of age.

**Fig. 1:**
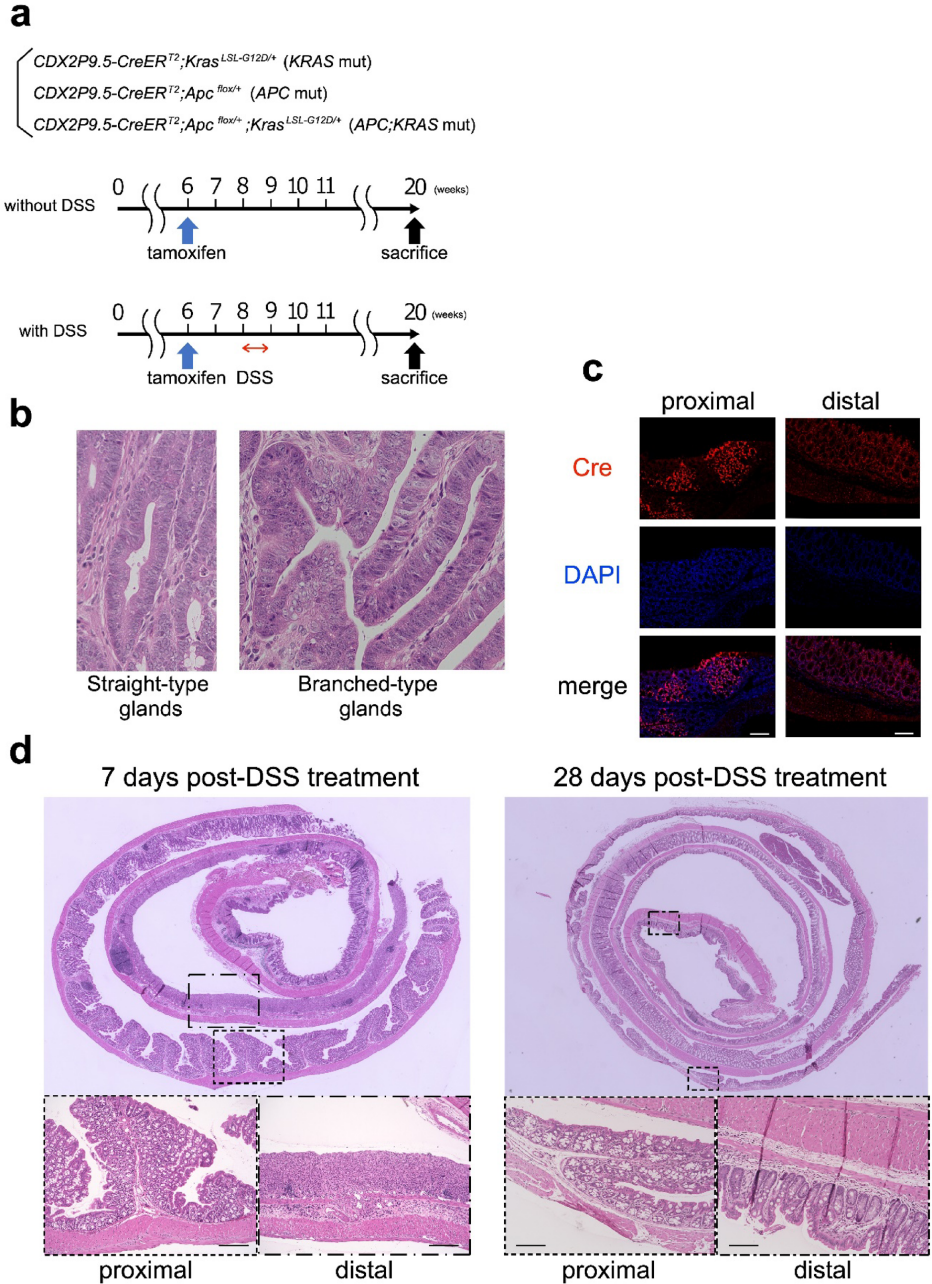
Experimental design, Cre expression, and inflammatory response to DSS treatment. (**a**) Schematic of the experimental design. “Without DSS” groups received tamoxifen at 6 weeks of age to induce Cre activity (*KRAS* : *n* = 7, *APC* : *n* = 6, *APC; KRAS* : *n* = 7). “With DSS” groups received tamoxifen at 6 weeks of age, followed by 1.5% DSS for 5 days at 8 weeks (*KRAS* : *n* = 7, *APC* : *n* = 9, *APC; KRAS*: *n* = 6). All mice were sacrificed at 20 weeks. (**b**) Typical examples of glandular morphology. Left: Straight-type glands, characterized by linear, simple structures. Right: Branched-type glands, showing complex, branching architecture. (**c**) Immunofluorescence staining revealed higher Cre expression in the proximal colon, with minimal expression in the distal colon (Scale bars: 200 μm). (**d**) H&E staining showed stronger DSS-induced inflammation in the distal colon, while the proximal colon exhibited only mild changes. Mice were sacrificed either 7 or 28 days after completion of DSS administration. By 28 days post-DSS, inflammation had completely resolved in both regions (Scale bars: 100 μm).

Acute colitis was induced in 8-week-old mice by administering filter-purified drinking water (Millipore Corp., Billerica, MA, USA) containing 1.5% (w/v) DSS (MW 36,000–50,000; MP Biomedicals, Solon, OH, USA) for 5 days. From day 5 onwards, animals were received regular drinking water (Fig. 1a). Body weights were recorded weekly. The number of mice administered drugs in this study was as follows:

*APC; KRAS* mut without DSS: 7, *APC* mut without DSS: 6, and *KRAS* mut without DSS: 7, *APC; KRAS* mut with DSS: 6, *APC* mutations with DSS: 9, and *KRAS* mut with DSS: 7.

At 20 weeks of age, the entire colorectum of each mouse was removed immediately after euthanization and flushed with ice-cold phosphate-buffered saline (PBS). The intestinal tissue was sliced longitudinally, and the location, number, and diameter of polyps in the colon were recorded. To evaluate tumor location, the colon was divided into two equal halves: the proximal and distal segments. The proximal colon was defined as the segment from the cecum to the midpoint of the colon, while the distal colon spanned from the midpoint to the rectum. Tumor number and size were assessed prior to formalin fixation. Polyps were counted, and their size was measured manually using a caliper under a dissecting microscope (Stemi DV4, ZEISS) without staining to enhance visibility. No imaging software was used for this evaluation. For histological analysis, specimens were fixed in 4% PFA. Tumor tissues were divided, with one part embedded in paraffin and the other part embedded in OCT compound (Sakura Finetek Japan, Tokyo, Japan), rapidly frozen, and stored at − 80 °C. A hemispherical shape was assumed when estimating polyp volume.

### Ribonucleic acid (RNA) sequencing

The tumors and normal tissues were dissected and mechanically disrupted using a homogenizer. Total RNA was extracted from the homogenates using the RNeasy Mini Kit (Qiagen, Hilden, Germany) according to the manufacturer’s protocol. Library construction and data processing were performed at the Beijing Genome Institute (Beijing, China). The library was sequenced using the DNBSEQG400RS platform, and high-quality reads were obtained. Sequence alignment was conducted using the GRCm38 mouse reference genome version GCF_000001635.26_GRCm38.p6 (https://www.ncbi.nlm.nih.gov/assembly/GCF_000001635.26; accessed on December 10, 2024). RNA integrity was assessed using an Agilent Bioanalyzer to obtain RNA Integrity Numbers (RIN). The Dr. Tom multiple omics data mining system (https://biosys.bgi.com, accessed on December 18, 2024; Beijing Genome Institute, Beijing, China) was used to identify relevant sequencing accuracy, mapping efficiency, differentially expressed genes (DEGs), and to perform enrichment analysis of the GO and Kyoto Encyclopedia of Genes and Genomes (KEGG) pathways. DEGs were identified using DEGseq^26^, which is suitable for small-sample RNA-seq analysis. DEGs were defined based on fold change thresholds (≥ 2.00) and adjusted P-values (≤ 0.001), calculated using the Benjamini–Hochberg method to control the false discovery rate.

### Histology and immunohistochemistry

FFPE sections were cut at 4 μm thickness, deparaffinized, rehydrated, and stained with H&E following standard protocols. Frozen sections were sectioned at a thickness of 7 μm using a cryostat. The sections were permeabilized with 0.1% Triton X-100 in PBS for 10 min, washed with PBS, and blocked with 5% bovine serum albumin for 1 h at room temperature. Immunostaining was performed using the following primary antibodies: anti-F4/80 (Cell Signaling, D2S9R, 1:400), anti-CD8 (Cell Signaling, D4W2Z, 1:100), anti-CD4 (Cell Signaling, D2D2Z, 1:100), anti-FOXP3 (Cell Signaling, D6O8R, 1:400) and anti-Ki67 antibody (GeneTex, GTX16667, 1:400). Incubation was carried out overnight at 4 °C, followed by treatment with a fluorescent secondary antibody (AF568, Invitrogen, A-11011, 1:250) for 1 h. The slides were counterstained with DAPI, mounted with an antifade medium.

Multiplex immunofluorescence staining was performed using the Opal Multiplex IHC Kit (Akoya Biosciences). Two sets of antibody combinations were applied as follows: one comprising anti-CD163 (Abcam, ab182422, 1:500) and anti-NOS2 (Santa Cruz Biotechnology, SC-651, 1:100), and the other consisting of anti-Cyclin D1 (Abcam, ab16663, 1:200) and anti-pERK1/2 (Cell Signaling, D13.14.4E, 1:200). The sections underwent sequential microwave treatment, antigen retrieval with citrate buffer (pH 6.0), and staining with specific opal fluorophores. For pERK1/2 staining, antigen retrieval was performed using buffer (pH 9.0) instead. After all staining steps, sections were counterstained with DAPI.

All fluorescence images were captured using a BZX710 fluorescence microscope (Keyence, Osaka, Japan) under standardized conditions, with fixed exposure time and light intensity settings for all samples. We analyzed 13 tumors in *APC; KRAS* mut mice without DSS and 15 tumors in *APC; KRAS* mut mice with DSS. For the comparison between DSS-treated *APC* and *APC; KRAS* mut mice, 14 tumors per group were analyzed. For each tumor, several representative fields were randomly selected at 200× magnification. The number of analyzed fields per tumor varied depending on sample quality and staining clarity, typically ranging from 1 to 3 per tumor. All images were captured from within the tumor tissue. Positive regions for each antibody were quantified by defining luminance thresholds using the Hybrid Cell Counting application (BZ-X analysis software, version 1.3.1.1; Keyence, Osaka, Japan). The same criteria and software settings were applied uniformly across all groups to minimize analytical variability.

### Histological classification of glandular architecture

To evaluate the architectural differences in tumor histology, we categorized tumor glands into the following two morphological types: Straight-type glands, defined as unbranched, linear structures; and Branched-type glands, defined as complex, multi-luminal glandular patterns with frequent branching (Fig. 1b).

Based on these criteria, we examined H&E-stained tumor sections and assessed the relative predominance of each gland type within individual tumors. Tumors were classified into the following two categories according to the dominant glandular pattern:

Well-organized Type Tumor: tumors where straight-type glands were predominant and.

Branched Type Tumor: tumors where branched-type glands were predominant.

Two investigators blinded to the genotype independently reviewed and classified tumors.

### Statistical analysis

All values were expressed as means ± standard deviations (SDs). Group comparisons were performed using Mann-Whitney U tests, χ^2^ tests, unpaired *t* test, or Fisher’s exact tests. Body weight was analyzed using Kruskal–Wallis tests at each time point to detect intergroup differences, followed by Steel–Dwass all-pairs post hoc tests when the initial test showed significant differences. Differences were considered statistically significant when *P* < 0.05. All statistical analyses were conducted using the JMP 10 software (SAS Institute Inc.).

## Results

### Tamoxifen-induced Cre expression and inflammation dynamics in the colon

Immunofluorescence staining for tamoxifen-induced Cre recombinase revealed its differential expression in the colon (Fig. 1c). Cre expression was predominantly observed in the proximal colon, whereas minimal expression was detected in the distal colon. This suggests that recombination efficiency and genetic modifications induced by Cre were more prominent in the proximal region. Histological analyses at various time points following DSS administration revealed distinct regional differences in inflammation (Fig. 1d). Seven days after DSS treatment, the distal colon exhibited significant inflammatory cell infiltration, whereas the proximal colon showed only minimal inflammation. By 28 days post-DSS treatment, the inflammatory response subsided in both regions, with no significant residual inflammatory changes observed. These findings indicate that the distal colon was more susceptible to DSS-induced acute inflammation, but the inflammatory effects were transient and resolved over time in both regions.

### Tumor formation *APC*,* KRAS*, and *APC; KRAS* mut mice with/without DSS

To assess the impact of *APC* and *KRAS* mutations on tumorigenesis, multiple mouse models were established, including *APC; KRAS* mut, *APC* mut, and *KRAS* mut. No macroscopically visible tumors were detected in *KRAS* and *APC* mut mice without DSS treatment. Furthermore, histologic evaluation confirmed the absence of tumor formation in these groups (Supplementary Fig. S1a,b). However, in *APC; KRAS* mut mice without DSS, small tumors were detected in the proximal colon.

**Fig. 2:**
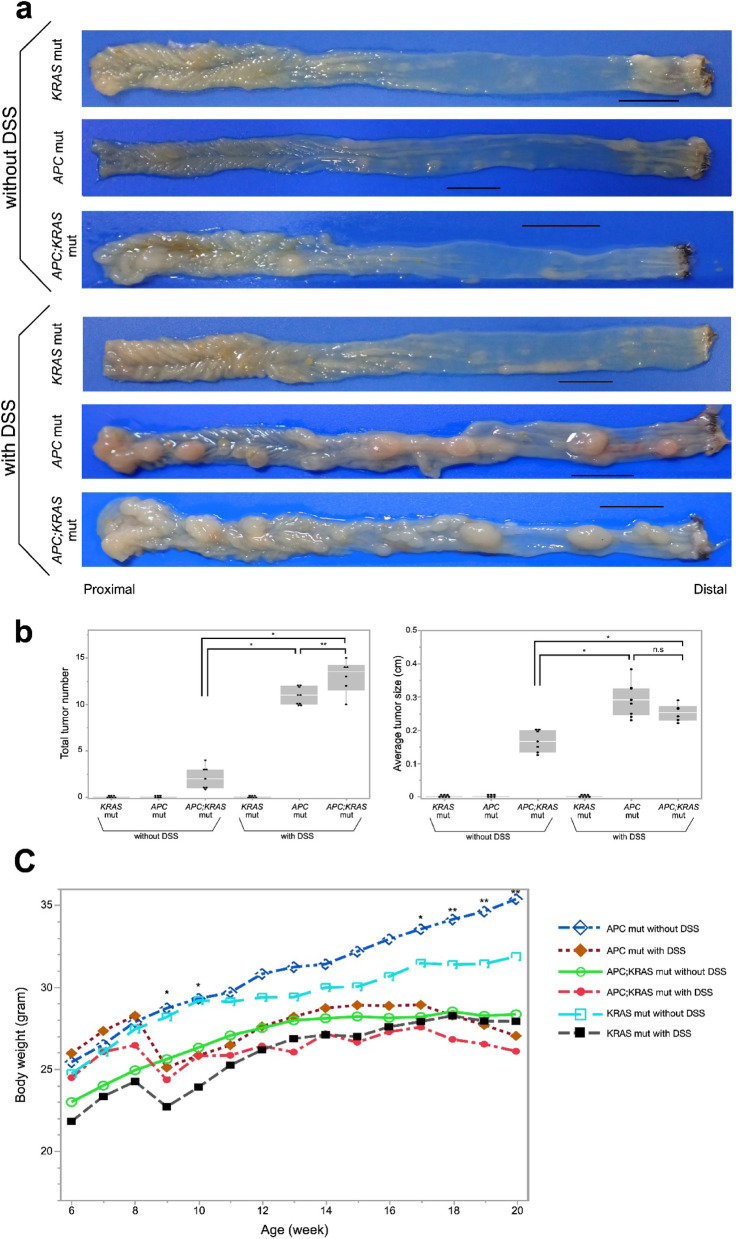
Macroscopic features of colons in different experimental groups. (**a**) Macroscopic views of colons from experimental mice showing tumor development across genotypes and DSS conditions. No tumors were observed in *KRAS* mut mice, regardless of DSS treatment. *APC* and *APC; KRAS* mut mice treated with DSS developed multiple tumors. In *APC; KRAS* mut mice without DSS, small tumors were observed in the proximal colon (Scale: 10 mm). (**b**) This figure summarizes the total tumor number and average tumor size per mouse in all experimental groups. *APC; KRAS* mut without DSS mice developed 2.1 ± 1.2 tumors (0.20 ± 0.01 cm). *APC* mut with DSS mice had 10.9 ± 0.9 tumors (0.31 ± 0.08 cm). *APC; KRAS* mut with DSS mice showed 13.0 ± 1.8 tumors (0.26 ± 0.02 cm). No tumors were observed in the other groups. Tumor number and size were significantly higher in DSS-treated mice with APC mutations. n.s.: not significant, *P < 0.01, **P < 0.05. (**c**) Longitudinal changes in average body weight from 6 to 20 weeks. DSS-treated groups exhibited transient weight loss around weeks 8–10 and slower recovery than untreated groups. APC and APC; KRAS with DSS showed persistent weight suppression by week 20. Statistical analysis revealed significant differences in body weight among the six groups at weeks 9, 10, 17, 18, 19, and 20 (*P* = 0.02, 0.03, 0.02, 0.02, < 0.01, and < 0.01, respectively) ; however, Steel–Dwass post hoc testing showed a significant difference only at week 20 between the *APC* with and without DSS groups. Each line represents the group mean. Group sizes: APC mut without DSS (*n* = 6), APC mut with DSS (*n* = 9), APC; KRAS mut without DSS (*n* = 7), APC; KRAS mut with DSS (*n* = 6), KRAS mut without DSS (*n* = 7), and KRAS mut with DSS (*n* = 7). *P < 0.01, **P < 0.05.

Following DSS administration, neither macroscopic nor histological tumors were detected in *KRAS* mut mice with DSS (Supplementary Fig. S1c), indicating that DSS exposure did not promote tumorigenesis in the presence of the *KRAS* mutation alone. In contrast, *APC* mut mice with DSS developed multiple tumors throughout the colon, predominantly in the distal region. A similar pattern was observed in *APC; KRAS* mut mice treated with DSS, which exhibited widespread tumor formation throughout the colon (Fig. 2a). We also summarized the total tumor number and average tumor size per mouse in the tumor-developing groups to provide a comprehensive overview (Fig. 2b). Tumor number significantly differed between *APC; KRAS* without DSS vs. *APC; KRAS* with DSS (*P* < 0.01), and vs. *APC* with DSS (*P* < 0.01); a modest difference was also seen between *APC; KRAS* with DSS and *APC* with DSS (*P* = 0.03). Tumor size also differed significantly between *APC; KRAS* without DSS and both DSS-treated groups (*P* < 0.01 and *P* < 0.01), but not between *APC; KRAS* with DSS and *APC* with DSS (*P* = 0.11).

To evaluate the physiological impact of DSS administration, body weight was monitored weekly. All groups exhibited gradual weight gain during the observation period. However, DSS-treated groups showed transient weight loss around 8–10 weeks, coinciding with DSS administration. Notably, *APC* with DSS and *APC; KRAS* with DSS groups exhibited a decreasing trend in body weight toward 20 weeks (Fig. 2b). Statistical analysis revealed significant differences in body weight among the six groups at multiple time points, particularly at weeks 9, 10, 17, 18, 19, and 20 (*P* = 0.02, 0.03, 0.02, 0.02, < 0.01, and < 0.01, respectively). However, post hoc Steel–Dwass tests identified a significant difference only between the *APC* mut with and without DSS groups at week 20.

Based on the findings in Fig. 1c,d, we determined that the proximal and distal colon should be evaluated separately, as Cre recombination was predominantly observed in the proximal colon, while DSS-induced inflammation was more prominent in the distal colon.

In the subsequent analyses, we first compared proximal tumors in *APC; KRAS* mut mice with and without DSS to investigate the impact of transient inflammation. We then examined distal tumors in DSS-treated *APC* and *APC; KRAS* mice to evaluate the role of *KRAS* mutation under inflammatory conditions. These comparisons enabled us to dissect the distinct contributions of inflammation and *KRAS* activation to tumor development.

### Comparison of proximal tumors in DSS-treated and untreated *APC; KRAS* mut mice

#### DSS treatment increases tumor size and number in the proximal colon

**Fig. 3:**
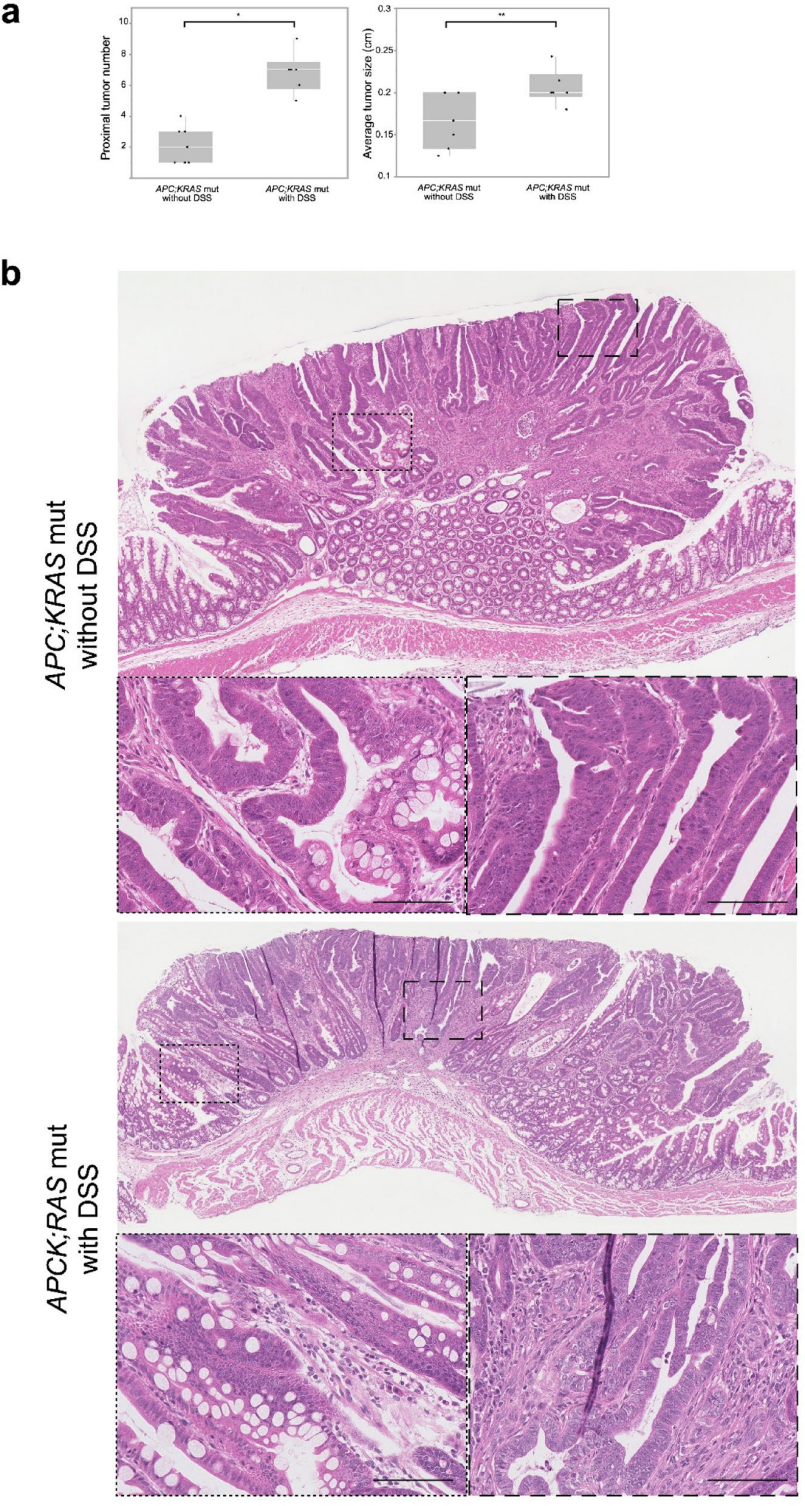
Polyp number, size and Histological overview in CDX2P9.5- *CreER*^*T2*^;*Apc*^*flox/+*^;*Kras*^*LSL−G12D/+*^ with and without DSS at the proximal colon. (**a**) This figure shows tumor burden (number and size) in the proximal colon, where DSS administration significantly increased both parameters, indicating that even mild inflammation can promote tumor progression in a genetically susceptible background. *P < 0.01, **P < 0.05. (**b**) H&E staining revealed that all tumors in both groups (nine tumors from *APC; KRAS* mice without DSS and 11 tumor with DSS) classified as Branched Type Tumor. (Scale: 100 μm).

*APC; KRAS* mut mice not treated with DSS, small tumors were observed in the proximal colon. Upon DSS administration, these mice developed a significantly higher number of tumors (6.8 ± 1.3 vs. 2.1 ± 1.2, *P* < 0.01) and increased tumor size (0.16 ± 0.03 vs. 0.21 ± 0.02 cm, *P* = 0.04) in the proximal colon compared to their untreated counterparts (Fig. 3a). H&E staining revealed that tumors in both DSS-treated and untreated *APC; KRAS* mut mice exhibited a similar branched tubular morphology, suggesting that DSS treatment did not alter the fundamental histological features of the tumors (Fig. 3b). Histological assessment was performed for nine tumors from untreated mice and 11 tumors from DSS-treated mice, all of which were classified as Branched Type Tumor based on glandular architecture.

#### DSS-associated transcriptomic changes in proximal colon tumors

All RNA samples used for library construction had RIN values ≥ 7, indicating high RNA integrity (Supplementary Table. S1). Sequence quality distribution is presented in Supplementary Fig. S2. Principal component analysis revealed moderate clustering of samples based on treatment status, with some overlap between groups (Supplementary Fig. S3).

**Fig. 4:**
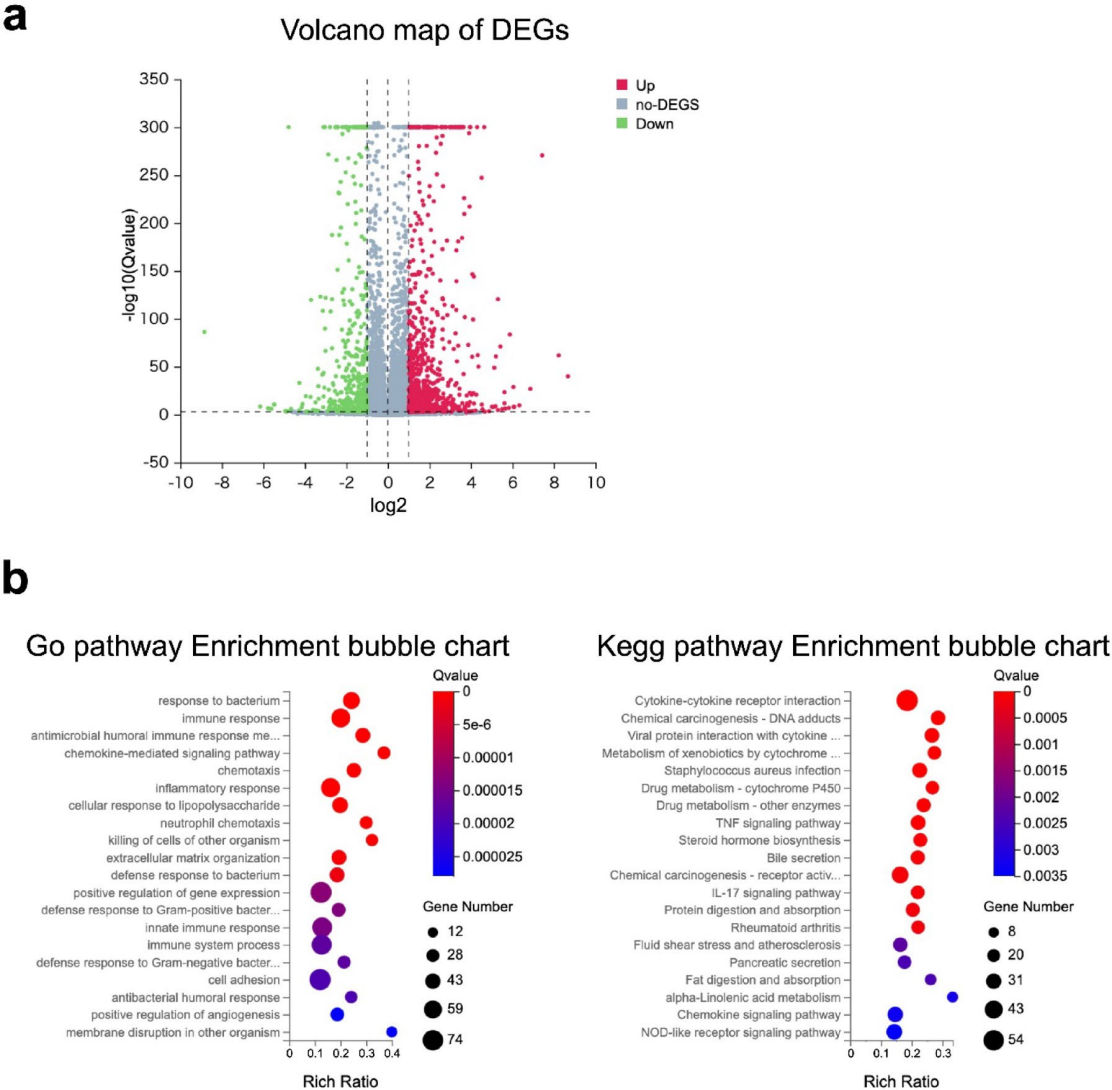
Volcano map of DEGs and Gene expression analysis of *CDX2P9.5- CreER*^*T2*^;*Apc*^*flox/+*^;*Kras*^*LSL−G12D/+*^ with and without DSS at proximal colon tumor. (**a**) Volcano plot showing DEGs identified between DSS-treated and untreated *APC; KRAS* mut tumors (*n* = 2 per group) based on adjusted P-values ≤ 0.001 and fold change ≥ 2.0. Red dots indicate significantly upregulated genes, and green dots indicate significantly downregulated genes. Several DEGs were detected despite the limited sample size; therefore, these findings should be interpreted with caution. (**b**) GO and KEGG pathway enrichment analyses, highlighting biological processes and molecular pathways that are differentially activated between DSS-treated and untreated tumors. The results suggest that DSS exposure modulates key signaling pathways relevant to tumor growth.

Volcano plot analysis identified several DEGs between DSS-treated and untreated *APC; KRAS* mut tumors (*n* = 2 per group, Fig. 4a). Due to the limited sample size, these transcriptomic findings should be interpreted cautiously. The analysis is considered exploratory and primarily hypothesis-generating, requiring further validation with larger cohorts. GO and KEGG enrichment analyses revealed multiple immune-related pathways (Fig. 4b). The complete list of DEGs is provided in Supplementary Table. S2.

#### No significant changes in immune cell infiltration by DSS in proximal tumors

To further investigate the immune microenvironment and cellular proliferation in proximal colon tumors, immunofluorescence staining was performed for F4/80, CD4, CD8, and Ki67 in *APC; KRAS* mut tumors, with and without DSS treatment.

**Fig. 5:**
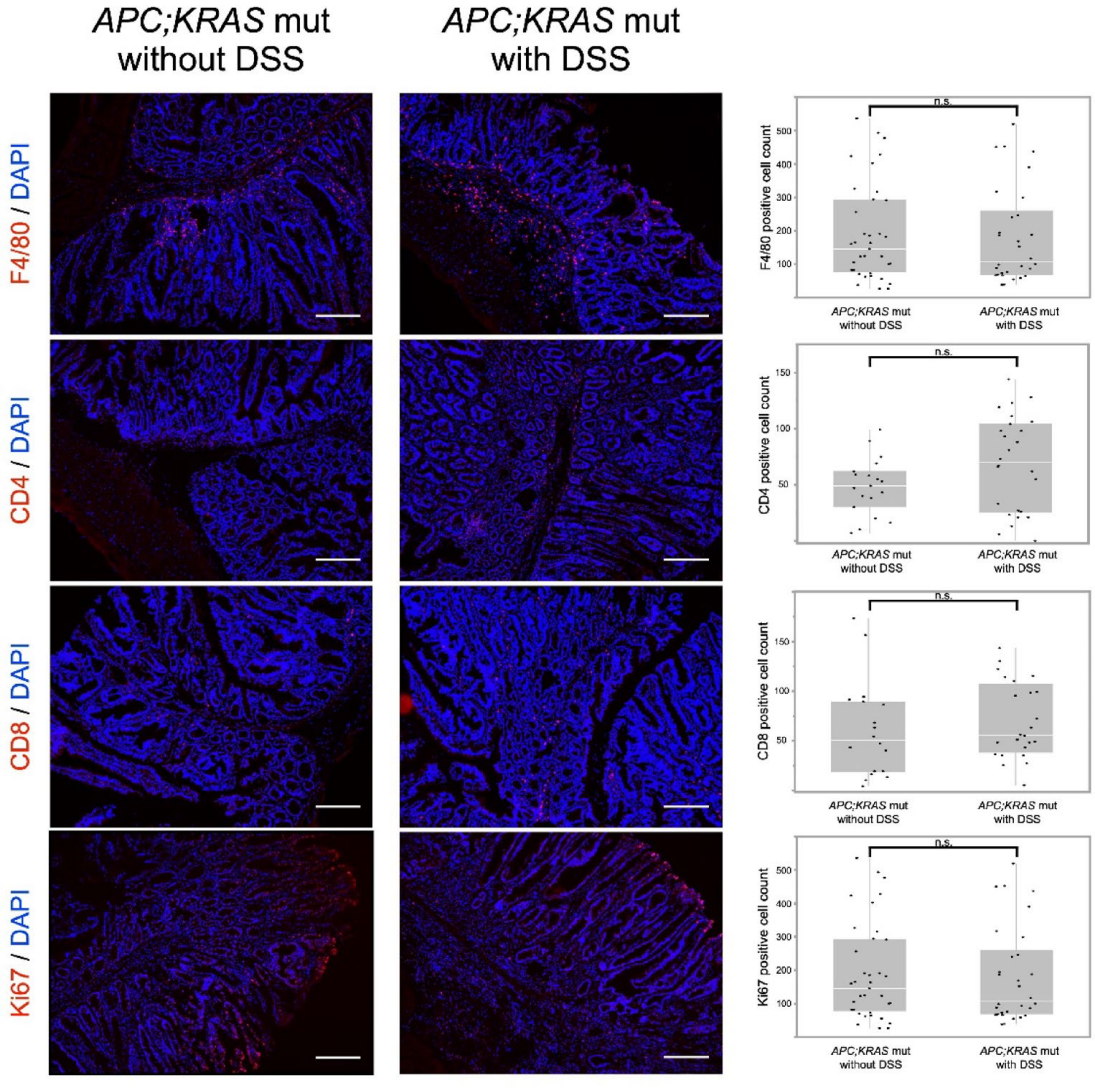
Immunofluorescence analysis of proximal colon tumors in *CDX2P9.5-CreER*^*T2*^;*Apc*^*flox/+*^;*Kras*^*LSL−G12D/+*^ with and without DSS. Immunofluorescence staining for F4/80, CD4, CD8, and Ki67 showed no significant differences in immune cell infiltration or proliferation between DSS-treated and untreated groups. Quantification fields: F4/80: 30 (DSS) vs. 36 (untreated), CD4: 26 vs. 19, CD8: 24 vs. 18, Ki67: 23 vs. 37. (Scale: 200 μm), n.s: not significant.

The results showed no significant differences in the expression levels of F4/80 (*APC; KRAS* mut with DSS vs. without DSS ;189.1 ± 145.7 vs. 180.7 ± 144.0, *P* = 0.38), CD4 (48.4 ± 25.2 vs. 68.7 ± 42.7, *P* = 0.07), CD8 (60.3 ± 48.3 vs. 69.8 ± 35.5, *P* = 0.13), or Ki67 (197.6 ± 107.9 vs. 188.6 ± 59.8, *P* = 0.40) between the two groups (Fig. 5). Quantitative analysis confirmed that DSS treatment did not significantly alter macrophage infiltration, T cell populations, or tumor cell proliferation in proximal colon tumors.

### Comparison of distal tumors in DSS-treated *APC; KRAS* and *APC* mut mice

#### Similar tumor burden but distinct histological features between genotypes

**Fig. 6:**
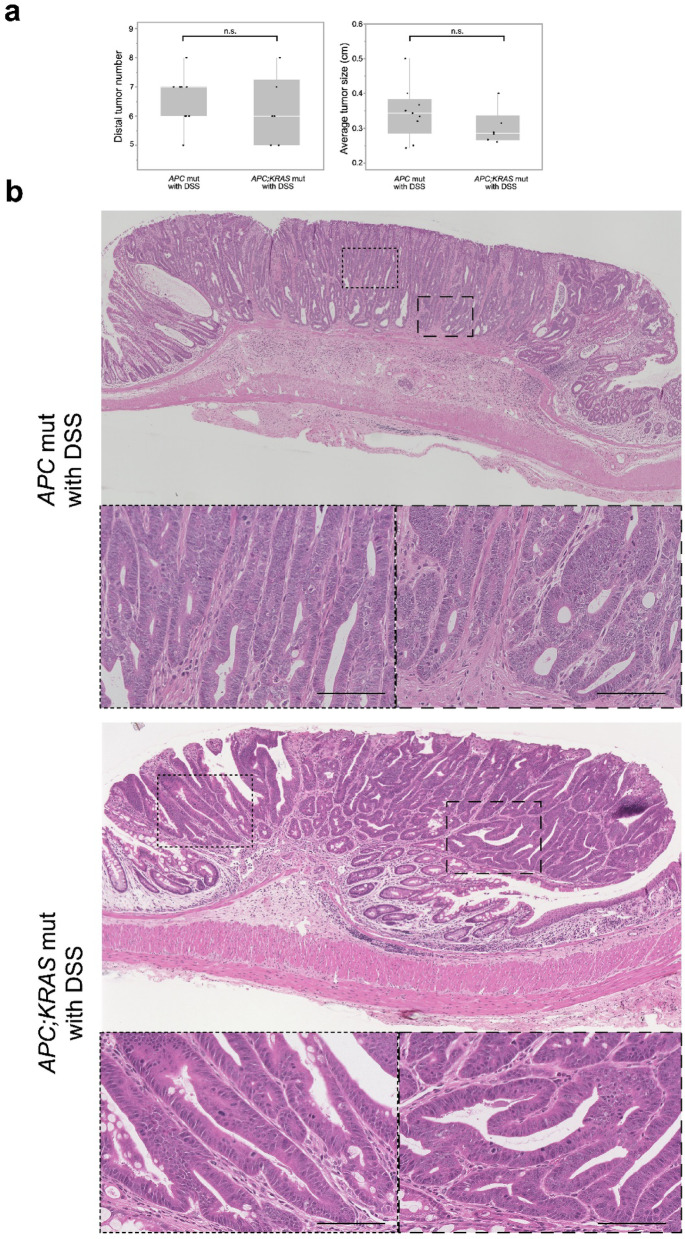
Polyp number, size, and histological overview in *CDX2P9.5-CreER*^*T2*^;*Apc*^*flox/+*^ with DSS vs. *CDX2P9.5-CreER*^*T2*^;*Apc*^*flox/+*^;*Kras*^*LSL−G12D/+*^ with DSS at the distal colon. (**a**)This figure presents a comparison between the polyp number and size in the distal colon between the *APC* mut and *APC*;*KRAS* mut mice, both treated with DSS. No significant differences were observed between the groups, n.s.: not significant. (**b**) H&E staining revealed distinct histological characteristics between the two genotypes. Among 13 tumors from *APC* mut mice, 11 and two were classified as Well-organized and Branched Type Tumors, respectively. All 11 tumors from *APC; KRAS* mut mice were Branched Type Tumor (*P* < 0.01). (Scale: 100 μm).

DSS-treated *APC* mut and *APC; KRAS* mut mice exhibited widespread tumor formation throughout the colon, predominantly in the distal region. The number (6.5 ± 0.9 vs. 6.2 ± 1.2, *P* = 0.23) and size (0.30 ± 0.05 vs. 0.34 ± 0.08 cm, *P* = 0.26) of distal colon polyps were not significantly different between the two genotypes (Fig. 6a). These tumors were derived from the same experimental cohort as those shown in Fig. 3a. Overall, 13 tumors from *APC* mut mice were analyzed, of which 11 and two were classified as Well-organized and Branched Type Tumors, respectively. In contrast, all 11 tumors from *APC; KRAS* mut mice were classified as Branched Type Tumor (Fig. 6b, *P* < 0.01).

#### *KRAS* mutation alters immune and ECM-related pathways in distal tumors

**Fig. 7:**
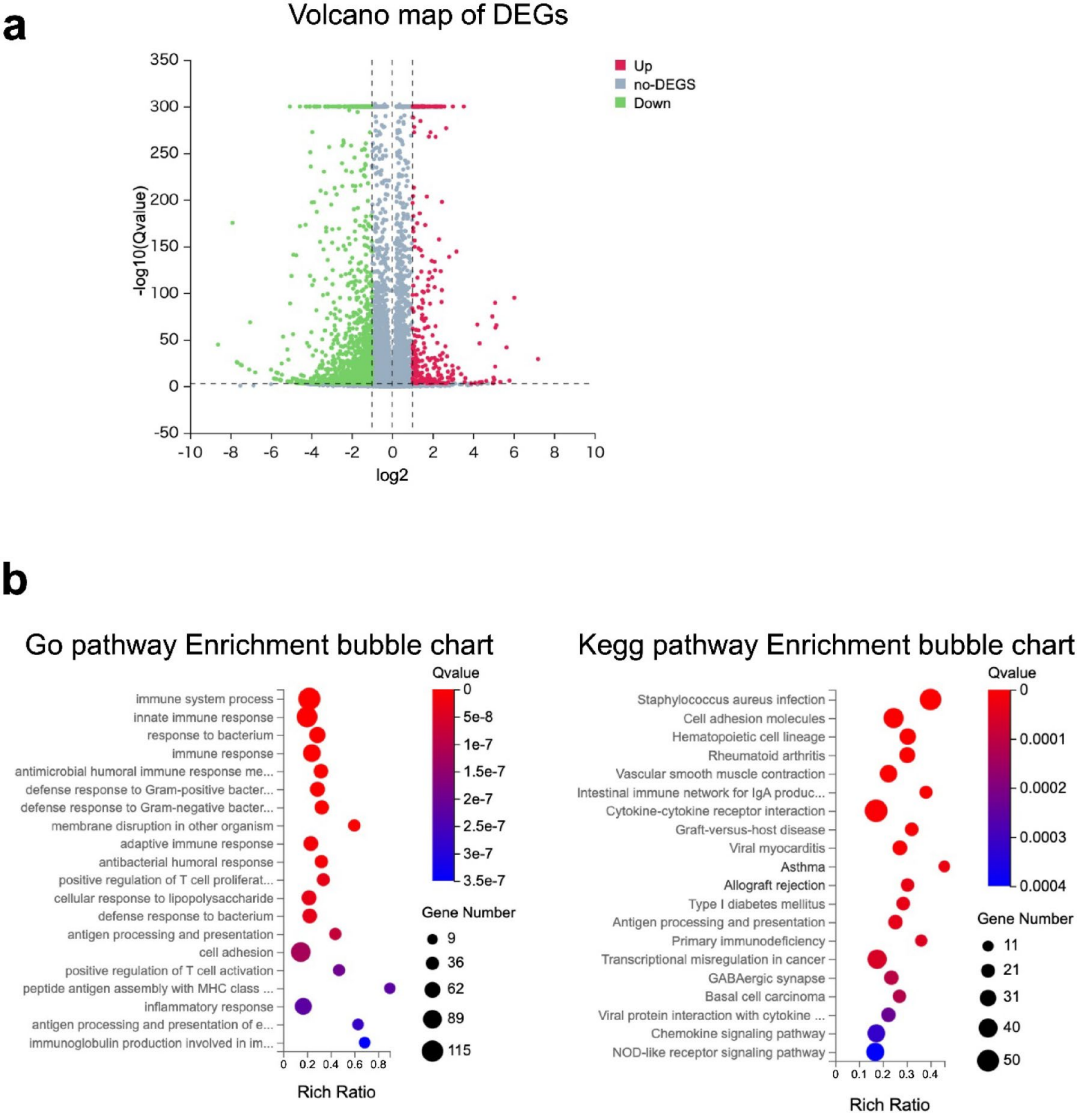
Volcano map of DEGs and gene expression analysis of *CDX2P9.5-CreER*^*T2*^;*Apc*^*flox/+*^ with DSS and *CDX2P9.5- CreER*^*T2*^;*Apc*^*flox/+*^;*Kras*^*LSL−G12D/+*^ with DSS at distal colon. (**a**) Volcano plot showing DEGs between *APC* mut tumors (*n* = 4) and *APC; KRAS* mut tumors (*n* = 3) with DSS. DEGs were selected based on fold change ≥ 2.00 and adjusted P-values (Q-value) ≤ 0.001. Red dots indicate significantly upregulated genes; green dots indicate significantly downregulated genes. (**b**) GO and KEGG pathway enrichment analyses of the identified DEGs. Enriched pathways included immune-related processes, extracellular matrix remodeling, and cytokine–cytokine receptor interaction, suggesting that *KRAS* mutations promote immunomodulatory and stromal remodeling pathways under inflammatory conditions.

Transcriptomic profiling indicated distinct differences in gene expression patterns between *APC* mut and *APC; KRAS* mut with DSS tumors. A total of 304 DEGs were identified between *APC* mut and *APC; KRAS* mut with DSS (fold change ≥ 2.00 and adjusted P-values ≤ 0.001, Fig. 7a), and the full list is provided in Supplementary Table S3. Enrichment analysis identified pathways associated with immune response, extracellular matrix remodeling, and cytokine signaling, suggesting that *KRAS* mutations contribute to distinct molecular changes in response to DSS-induced inflammation (Fig. 7b).

#### Increased Tregs and M2 macrophages in *APC; KRAS* mut tumors suggest a suppressive TME

**Fig. 8:**
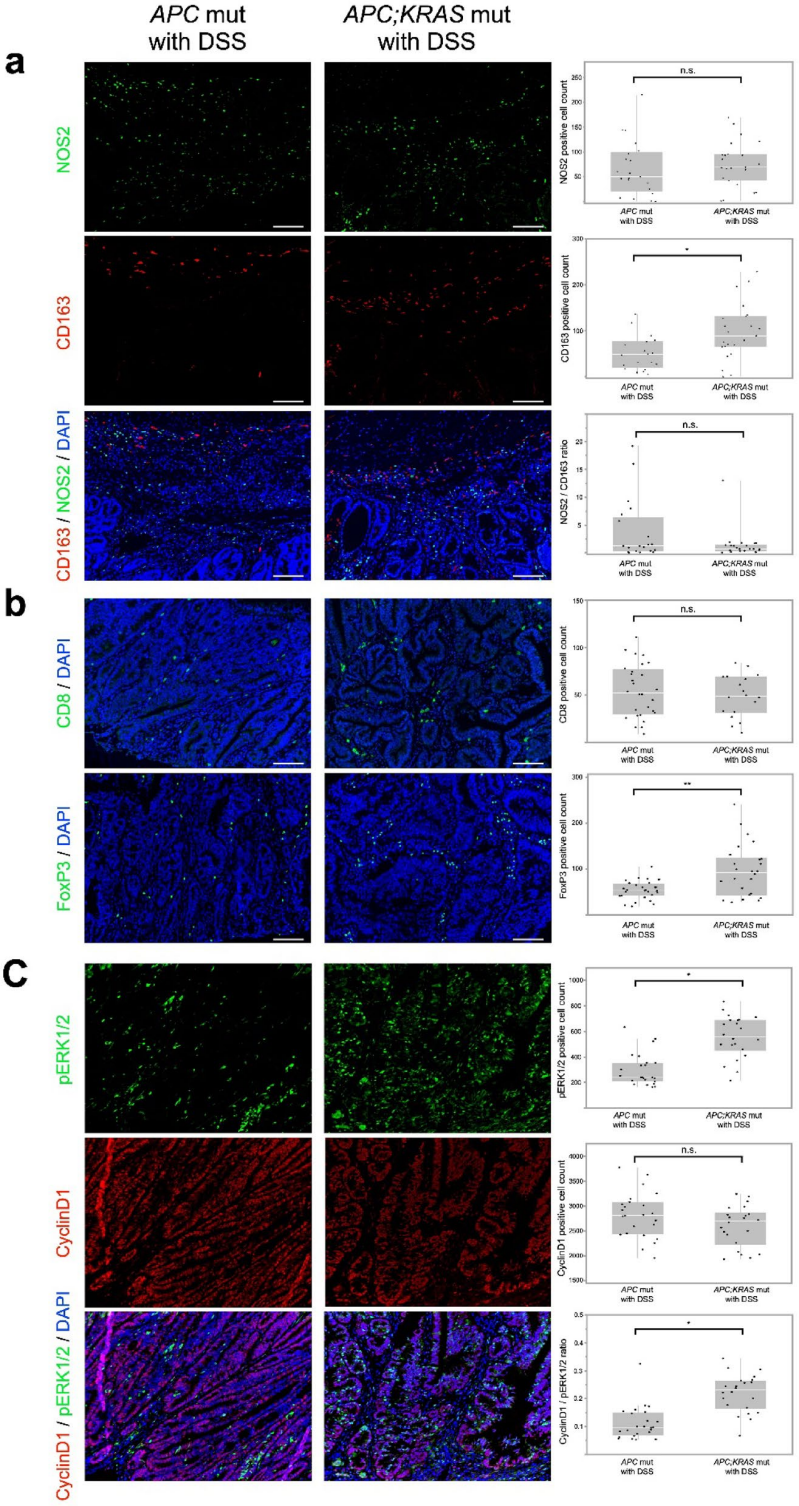
Immunofluorescence analysis of *CDX2P9.5*-*CreER*^*T2*^;*Apc*^*flox/+*^ mut with DSS and *CDX2P9.5-CreER*^*T2*^;*Apc*^*flox/+*^;*Kras*^*LSL−G12D/+*^ with DSS tumors. (**a**) Immunofluorescence staining for NOS2 and CD163 showed an increased number of M2 macrophages (CD163-positive) in *APC; KRAS* mut tumors compared with *APC* mut tumors, whereas the M1/M2 ratio did not significantly differ. Quantification fields: NOS2 and CD163: 21 fields (*APC*) vs. 22 fields (*APC; KRAS*). (**b**) Staining for CD8 and FOXP3 demonstrated no significant difference in CD8-positive T cells, while FOXP3-positive Tregs were significantly more abundant in *APC; KRAS* mut tumors, indicating an immunosuppressive tumor microenvironment. Quantification fields: CD8: 28 fields (*APC*) vs. 18 fields (*APC; KRAS*); FOXP3: 28 vs. 16. (**c**) Dual immunofluorescence staining for pERK1/2 and CyclinD1 revealed similar CyclinD1 expression between groups, but significantly higher pERK1/2 expression in *APC; KRAS* mut tumors. The ratio of pERK1/2 to CyclinD1-positive cells was also greater in *APC; KRAS* mut tumors. Quantification fields: pERK1/2 and CyclinD1: 22 fields (*APC*) vs. 24 fields (*APC; KRAS*). Scale: 200 μm, n.s.: not significant, *P < 0.01, **P < 0.05.

Tumor microenvironment analysis showed genotype-specific differences in immune cell populations. Macrophage analysis showed an increased presence of M2 macrophages (CD163-positive) in *APC; KRAS* mut tumors (*APC* mut vs. *APC; KRAS* mut : 56.9 ± 11.7 vs. 95.5 ± 11.2, *P* = 0.01); however, the M1/M2 ratio did not differ significantly between the two genotypes (3.6 ± 0.92 vs. 1.4 ± 0.91,*P* = 0.09), indicating that both inflammatory and immunosuppressive macrophage populations coexisted within the tumor microenvironment (Fig. 8a). T-cell analysis revealed no significant differences in CD8-positive T-cell counts between the genotypes (54.1 ± 28.3 vs. 48.8 ± 22.4, *P* = 0.50). However, the number of FOXP3-positive regulatory T cells (Tregs) was significantly higher in *APC; KRAS* mut tumors than those of *APC* (53.5 ± 20.3 vs. 94.8 ± 56.7, *P* < 0.01) (Fig. 8b). These findings suggest that *KRAS* mutations influence the tumor microenvironment by modulating stromal and immune cell populations, particularly under inflammatory conditions. Dual immunofluorescence staining for CyclinD1 and phosphorylated ERK1/2 (pERK1/2) showed no difference in CyclinD1 expression between groups (2804.6 ± 456.9 vs. 2591.0 ± 406.9, *P* = 0.14), whereas pERK1/2 expression was higher in *APC; KRAS* mut tumors (296.9 ± 127.4 vs. 556.3 ± 166.1, *P* < 0.01). The ratio of pERK1/2 to CyclinD1-positive cells was significantly greater in *APC; KRAS* mut tumors than in *APC* mut tumors (0.11 ± 0.06 vs. 0.22 ± 0.07, *P* < 0.01) (Fig. 8c).

## Discussion

This study examined the impact of inflammation and genetic mutations on tumor development in a mouse model harboring *APC*, *KRAS*, or combined *APC; KRAS* mutations. The results showed that, in the absence of DSS, no tumors developed in the distal colon in either the *APC* mut or *APC; KRAS* mut groups. However, upon DSS administration, multiple tumors formed in the distal colon tumors, and *APC; KRAS* mut mice developed small proximal colon tumors even without DSS treatment, with both tumor size and number increasing upon DSS administration. Furthermore, *KRAS* mutations alone did not result in tumor formation, regardless of DSS treatment.

One key finding is that tumorigenesis risk is not limited to chronic or severe inflammation, such as ulcerative colitis, but that even transient and mild inflammation can promote tumor formation and growth. These results underscore the importance of inflammation control in cancer prevention, suggesting that early management of inflammation may reduce the risk of tumor development. This may also be an effective strategy for cancer prevention even in individuals without chronic inflammation.

Several mechanisms may underlie the tumorigenic effects observed due to mild inflammation in this study. A possibility is that mild inflammation may contribute to loss of heterozygosity (LOH) in *APC*, thereby enhancing tumorigenesis. A previous study using *Apc*^*Min/+*^ mice reported that DSS administration caused the loss of the wild-type *APC* allele, which subsequently promoted tumor formation^27^. This suggests that the inflammatory environment following DSS administration might promote *APC* allele loss, resulting in increased genetic instability and enhanced tumor formation. Notably, DSS-induced inflammation was predominantly observed in the distal colon, where tumor formation was most pronounced. Several factors might have contributed to this observation. First, DSS has a stronger toxic effect on epithelial cells in the distal colon compared to the proximal colon, leading to greater tissue damage and localized inflammation. Second, the distal colon has a higher bacterial density, and DSS-induced barrier dysfunction may allow bacteria and their metabolites to penetrate the mucosa, further stimulating inflammation^28,29^. Consequently, inflammation in the distal colon may have triggered *APC* LOH, promoting tumor development.

Additionally, transient increases in inflammatory cytokines such as IL-6 and IL-1β following DSS administration have been reported, and these cytokines are known to promote tumor cell proliferation, immune evasion, and defective DNA repair, thereby facilitating tumorigenesis^30–32^. The transient action of these cytokines may have contributed to the creation of an environment conducive to tumor development. Moreover, DSS-induced transient disruption of the intestinal barrier^33^ allows bacterial infiltration and exposure of bacterial metabolites such as lipopolysaccharides to the intestinal mucosa, which activates immune responses and further contributes to tumorigenesis. This immune response likely involves activation of tumor-associated macrophages and cancer-associated fibroblasts, leading to a tumor-promoting TME.

Immunohistochemical analysis revealed no significant differences in immune cell infiltration or proliferation markers between proximal colon tumors in *APC; KRAS* mutants without DSS. This finding aligns with previous studies suggesting that DSS-induced inflammation is transient^27^. Consequently, the inflammatory response likely subsided by the time the tumors were harvested, resulting in minimal immune cell accumulation. In addition, the inflammatory response induced by DSS may not have reached the threshold required to trigger T cell infiltration.

A comparison between *APC; KRAS* mutants with DSS and *APC* mutants with DSS in the distal colon revealed that tumors harboring *KRAS* mutations exhibited a more immunosuppressive TME. Specifically, an increase in M2 macrophages and Tregs was observed, consistent with previous studies reporting similar immunosuppressive environments^34–37^. *KRAS* mutations have been suggested to drive immunosuppressive changes in the TME through pathways such as CXCL12-CXCR4 signaling and TGF-β signaling^38,39^. The findings of this study support this notion, further demonstrating the role of *KRAS* mutations in shaping the immune landscape of tumors. *APC* mutations play a central role in colorectal tumorigenesis, as evidenced by their high prevalence in colorectal cancer. Individuals with familial adenomatous polyposis, involving germline *APC* mutations, develop multiple adenomas. In this study, frequent tumor formation in *APC*-mutant mice indicates that *APC* mutation is a critical early event in tumorigenesis. Conversely, *KRAS* mutations alone did not lead to tumor formation regardless of DSS administration, suggesting that *KRAS* mutations alone are insufficient for tumorigenesis. While *KRAS* mutations activate proliferative signaling pathways, tumor formation appears to require additional genetic alterations such as *APC* mutations, which disrupt tumor suppressor mechanisms. These findings suggest that *APC* mutations create a permissive environment in which *KRAS* mutations exert tumor-promoting effects.

Furthermore, tumor localization is influenced by inflammation and Cre expression. In the absence of DSS, tumors predominantly developed in the proximal colon, whereas DSS exposure led to tumor formation in the distal colon. This pattern is likely due to the preferential induction of inflammation in the distal colon by DSS, as well as the higher Cre expression in the proximal colon under the *CDX2P9.5-CreERT2* system. Given that Cre recombination efficiency was higher in the proximal colon, the enhanced tumor formation in this region may be partly attributed to more efficient gene recombination, independent of DSS-induced inflammation. To minimize concerns about experimental variability, we note that tumors analyzed in both proximal (Fig. 3a) and distal regions (Fig. 6a) were obtained from the same experimental cohort; therefore, differences in tumor size are unlikely to result from experimental variability. Instead, these differences likely reflect region-specific tumor growth dynamics driven by distinct local microenvironments. These findings suggest that tumor development is shaped by a complex interplay between genetic mutations and inflammation, with tumor location and growth patterns determined by factors such as the extent and localization of inflammation and the distribution of genetic alterations.

The mouse model developed in this study serves as a powerful tool to investigate the roles of genetic mutations and inflammation in human tumorigenesis. The tumors observed in this model resembled adenocarcinomas originating from the adenoma-carcinoma sequence in *APC* mut mice. This model provides a valuable framework for exploring the impact of mild inflammation and evaluating therapeutic strategies, including approaches targeting M2 macrophage polarization, Treg suppression, CXCR4 inhibition, and immune checkpoint blockade. Using this model, researchers can assess treatment efficacy and gain comprehensive insights into how inflammation and TME alterations influence therapeutic outcomes.

In addition to transcriptomic alterations, we also observed distinct histological features between *APC* and *APC; KRAS* mut tumors in the distal colon. Specifically, *APC* tumors exhibited uniformly well-organized glandular architecture, whereas *APC; KRAS* tumors frequently showed branched tubular morphology. While direct evidence linking *KRAS* activation to “branched tubular” architecture is limited, it is known that *KRAS* activation can induce hyperplastic and serrated morphological features in the intestinal epithelium^40,41^. Although the architectural changes observed in our study cannot be definitively categorized as hyperplastic or serrated, these findings indicate that they may reflect a structural consequence of oncogenic *KRAS* signaling. While we could not directly demonstrate a causal relationship between *KRAS* mutations and the observed glandular architecture, the increased expression of pERK1/2 in *APC; KRAS* tumors compared to *APC* tumors may be consistent with the possibility that *KRAS* signaling contributes, at least partly, to the morphological alterations.

Despite its significance, this study had some limitations. First, although mild inflammation promotes tumorigenesis, the precise underlying mechanisms remain unclear. For instance, while this study suggests that inflammation may induce LOH in *APC*, direct validation is required. Further studies are needed to clarify the roles of gut microbiota, specific inflammatory cytokines, and epithelial damage/repair processes in tumorigenesis. In particular, experiments using controlled gut microbiota models are essential for assessing the effects of microbial metabolites. Furthermore, the mouse model used in this study was tailored to *APC* and *KRAS* mutations in colorectal cancer, limiting the applicability of the findings to other genetic alterations or tumor types. Moreover, although this model closely mimics human TME dynamics, discrepancies in immune cell composition and function may necessitate careful interpretation when translating these findings to clinical applications. Moreover, inflammation was induced through a single DSS administration in this study. Future research should investigate whether similar results can be obtained using different inflammatory stimuli, such as microbiota modulation or chemical insults. Finally, transcriptomic analysis was limited by the small sample size (*n* = 2 per group), resulting in reduced statistical power. Therefore, these findings should be interpreted with caution and considered exploratory and hypothesis-generating.

One additional limitation of our immunofluorescence analysis is the variability in the number of analyzed fields per tumor. Although we aimed to include multiple representative regions per tumor, the number of fields varied depending on the staining quality and tissue integrity, which may have introduced some bias. However, the fields were selected randomly under standardized imaging conditions, and the analysis was performed using the same thresholding parameters across all groups to minimize variability. Further studies with more uniform sampling strategies are warranted to validate these findings.

In conclusion, this study reveals that even mild inflammation can initiate tumorigenesis in *APC* mutant mice. Notably, a single low-dose DSS administration led to the formation of multiple tumors in the distal colon, as well as increased the tumor size and number in the proximal colon. These findings strongly suggest the significant role transient inflammation plays in promoting tumor development, underscoring the importance of understanding the interplay between genetic mutations and inflammation in colorectal carcinogenesis.

## Supplementary Information

Below is the link to the electronic supplementary material.

Supplementary Material 1

## Data Availability

Raw RNA-seq data have been deposited in the DDBJ Sequence Read Archive (DRA) under BioProject accession number PRJDB20745. The datasets generated and/or analyzed during the current study are available in the DDBJ repository (https://ddbj.nig.ac.jp/resource/bioproject/PRJDB20745). Other supporting data are available from the corresponding author Y.U. upon reasonable request.
