# Supplementary material for: Single-dose DSS-induced inflammation enhances colorectal tumorigenesis in APC and KRAS mutant mice
Source: Sci Rep. 2025 Nov 24;15:41623. doi: 10.1038/s41598-025-25577-1 (PMC12644814; doi:10.1038/s41598-025-25577-1)
Supplement: Supplementary file 1 — Supplementary Material 1 [file 41598_2025_25577_MOESM1_ESM.pdf]

Single-dose DSS-induced inflammation enhances colorectal tumorigenesis in *APC* and *KRAS* mutant mice

Kazuki Ishibashi[1], Yuji Urabe[1], Takahiro Uda[1], Yukiko Sako[1], Tomoyuki Gurita[1] Satoshi Masuda[1], Yoshiki Hatsushika[1], Takeo Nakamura[1], Hirona Konishi[1], Akiyoshi Tsuboi[1], Hidenori Tanaka[1], Ken Yamashita[1], Yoshihiro Kishida[1], Yuichi Hiyama[1], Hidehiko Takigawa[1], Toshio Kuwai[2], Hiroaki Niitsu[3], Takao Hinoi[3], Shiro Oka[1]

[1]Department of Gastroenterology, Graduate School of Biomedical and Health Sciences, Hiroshima University, Hiroshima, Japan

[2]Department of Gastrointestinal Endoscopy and Medicine, Hiroshima University Hospital, Hiroshima, Japan

[3]Department of Clinical and Molecular Genetics, Hiroshima University Hospital, Hiroshima, Japan

Supplementary Fig.S1

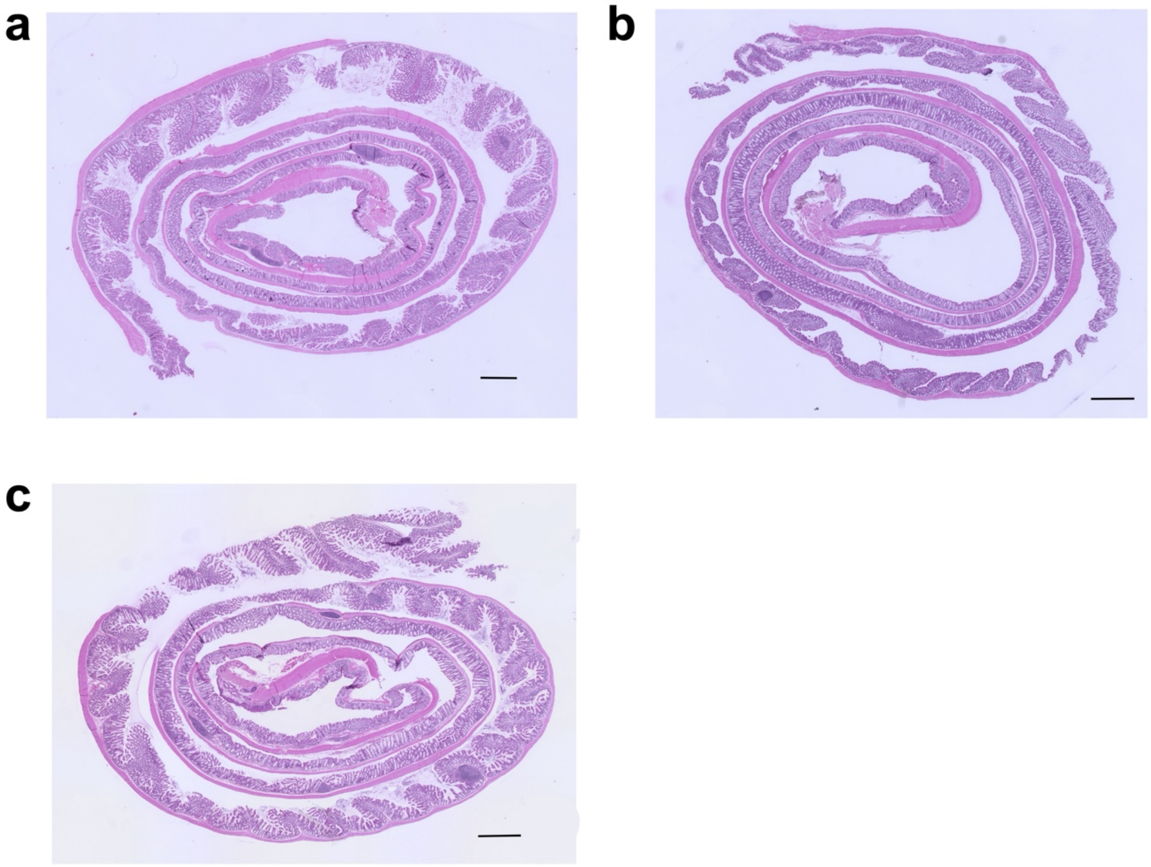

Supplementary Figure. S1: H&E images of *KRAS* mut mice with and without DSS, and *APC* mut mice without DSS.

(a) *KRAS* mut mice without DSS treatment showed no histologic evidence of neoplasia.

(b) *APC* mut mice without DSS treatment also demonstrated normal histology without tumor formation.

(c) *KRAS* mut mice with DSS treatment exhibited no tumorigenesis.

Scale bars: 1000 µm.

Supplementary Fig.S2

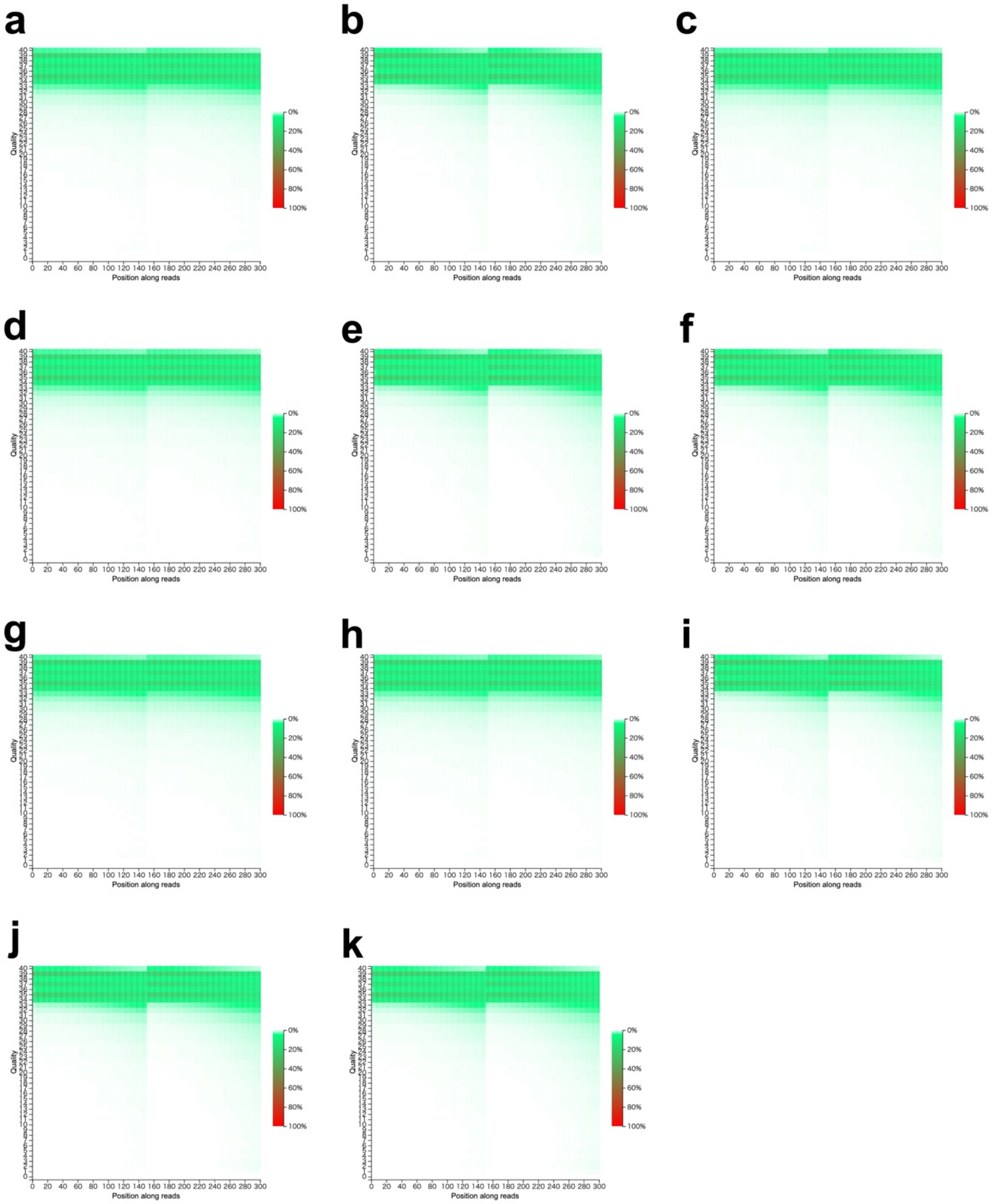

Supplementary Fig. S2: Base quality distribution across sequencing reads.

The X-axis represents the position of bases in reads, and the Y-axis represents the quality value of bases. Each point represents the number of bases reaching a certain quality value at each position, with darker colors indicating higher counts. Typically, base quality is lower at the start and end of reads, but overall, the low proportion of bases with quality scores <20 indicates high sequencing quality.

Panels a–k correspond to RNA-seq samples A–K as listed in Supplementary Table.S1

Supplementary Fig.S3

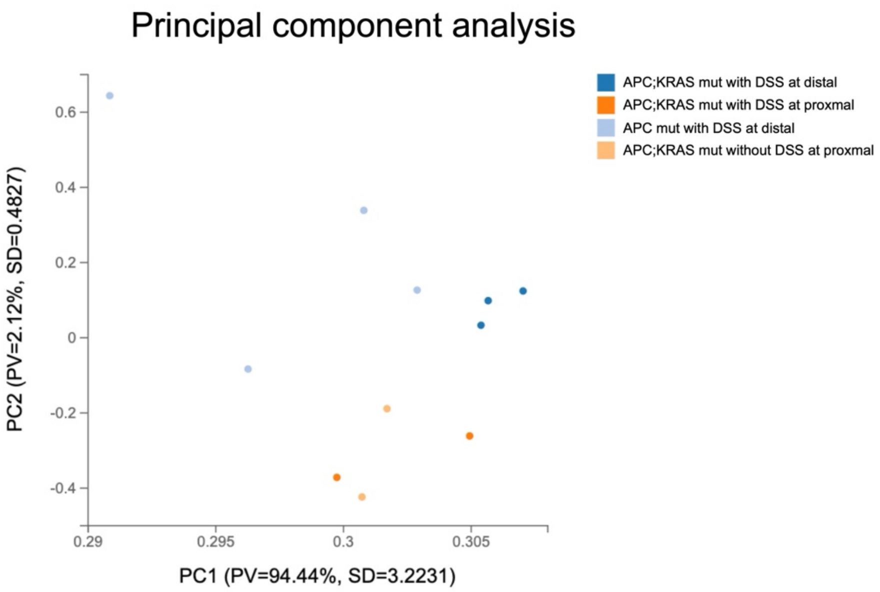

Supplementary Fig. S3: Principal Component Analysis (PCA)

PCA reduces multidimensional gene expression data into principal components to evaluate sample clustering. The X and Y axes represent the first two principal components, and the numbers in parentheses indicate the percentage of variance explained. Each point represents a sample, color-coded by group. PV: Proportion of variance; SD: Standard deviation.

Supplementary tables

Supplementary Table. S1: Summary of sequencing and mapping quality metrics for RNA-seq samples.

Sample No.	Genomic type and DSS	distal or proximal	RINe	Total Raw Reads (M)	Total Clean Reads (M)	Total Clean Bases (Gb)	Clean Reads Q20 (%)	Clean Reads Q30 (%)	Clean Reads Ratio (%)	Total Clean Reads (M)	Total Mapping(%)	Uniquely Mapping(%)
A	*APC;KRAS* mut without DSS	proximal	9	24.47	22.88	6.86	98.04	93.25	93.5	45.76	97.63	88.46
B	*APC* mut with DSS	distal	8.6	24.47	22.72	6.82	98.07	94.02	92.85	45.44	97.42	87.35
C	*APC;KRAS* mut with DSS	distal	8.4	24.47	22.65	6.79	98.03	93.21	92.56	45.29	97.32	88.15
D	*APC* mut with DSS	distal	7.2	25.34	23.29	6.99	98.18	93.71	91.91	46.58	97.16	85.7
E	*APC* mut with DSS	distal	7.9	24.47	22.69	6.81	98.08	94.04	92.73	45.39	97.33	87.7
F	*APC;KRAS* mut without DSS	proximal	9.5	23.59	22.14	6.64	98.06	93.97	93.85	44.28	97.76	89.89
G	*APC* mut with DSS	distal	9.3	24.47	22.98	6.9	97.93	92.85	93.91	45.97	97.65	90.38
H	*APC;KRAS* mut with DSS	distal	8.1	23.59	22.18	6.65	98.16	93.64	94.02	44.36	97.99	89.85
I	*APC;KRAS* mut with DSS	proximal	8.6	23.59	22.34	6.7	98.13	94.19	94.7	44.69	97.91	88.59
J	*APC;KRAS* mut with DSS	distal	7.9	23.59	22.02	6.61	98.03	93.94	93.34	44.03	97.61	87.44
K	*APC;KRAS* mut with DSS	proximal	8.7	23.59	22.51	6.75	97.95	93.69	95.42	45.03	97.97	89.84

Total Raw Reads (M): Number of reads before filtering (in millions)

Total Clean Reads (M): Number of reads after filtering (in millions)

Total Clean Bases (Gb): Number of bases after filtering (in Gb)

Clean Reads Q20 (%): Proportion of reads with quality greater than Q20 after filtering

Clean Reads Q30 (%): Proportion of reads with quality greater than Q30 after filtering

Clean Reads Ratio (%): Proportion of reads after filtering.

Total Clean Reads (M): Number of filtered reads (in millions)

Total Mapping (%): Proportion of clean reads mapped to the reference genome

Supplementary Table. S2: List of DEGs Between *APC;KRAS* Mut Tumors With and Without DSS Treatment

Gene ID	Gene Symbol	*APC;KRAS* with DSS Average Read Count	*APC;KRAS* without DSS Average Read Count	log2	Qvalue
100038862	'Btnl1'	125.4	14.71	-3.09168	2.26E-04
100042514	'Sprr2a3'	42827.63	19806.35	-1.11258	0.00388
102022	'Ces2a'	317.4	35.19	-3.17310	2.68E-06
102635496	'Malrd1'	121.8	13.35	-3.18922	0.02305
104174	'Gldc'	8.38	143.38	4.09610	0.00223
105005	'Lratd1'	1744.88	716.05	-1.28499	0.02772
108052	'Slc14a1'	37.34	385.33	3.36713	0.02214
108079	'Prkaa2'	1243.85	619.38	-1.00591	0.05000
108105	'B3gnt5'	265.52	689.1	1.37587	0.00182
108112	'Eif4ebp3'	843.48	223.52	-1.91594	0.01702
109254	'Adtrp'	642.05	97.03	-2.72613	5.95E-06
109791	'Clps'	13.01	235.87	4.17986	0.03457
110877	'Slc18a1'	76.89	369.53	2.26490	0.00378
115488029	'LOC115488029'	129.12	1027.23	2.99202	0.00000
11551	'Adra2a'	349.18	84.43	-2.04811	0.01220
11727	'Ang'	35.24	231.35	2.71500	0.01633
11813	'Apoc2'	597.62	133.25	-2.16514	0.01174
118568152	'LOC118568152'	24.22	133.38	2.46137	0.00754
118568683	'LOC118568683'	508.83	2037.36	2.00144	0.00000
118568705	'LOC118568705'	2949.44	13053.07	2.14588	0.00010
118568792	'LOC118568792'	80.94	417.23	2.36592	0.00278
12346	'Car1'	49260.89	9192.97	-2.42184	4.98E-40
12351	'Car4'	334.62	74.68	-2.16368	4.06E-05
12520	'Cd81'	3548.44	7928.35	1.15983	0.01108
12554	'Cdh13'	656.03	1855.64	1.50008	0.01660
12577	'Cdkn1c'	168.71	1012.01	2.58456	0.01295
12583	'Cdo1'	46.19	472.29	3.35391	9.85E-04
12841	'Col9a3'	47.08	302.93	2.68568	0.01965
12985	'Csf3'	0.98	49.55	5.66117	0.03230

13105	'Cyp2d9'	108.74	17.72	-2.61756	0.03634
13113	'Cyp3a13'	877.83	217.92	-2.01017	0.00109
13616	'Edn3'	261.22	32.38	-3.01217	2.86E-05
13730	'Emp1'	39491.88	18147.85	-1.12176	1.44E-07
140743	'Rem2'	97.23	342.52	1.81679	0.00110
14079	'Fabp2'	2838.28	342.92	-3.04908	1.14E-09
14114	'Fbln1'	437.71	1791.14	2.03283	0.01049
14263	'Fmo5'	237.72	35.61	-2.73896	0.00378
14734	'Gpc3'	371.14	44.67	-3.05457	4.70E-04
14776	'Gpx2'	39184.59	91193.66	1.21865	0.00145
14825	'Cxcl1'	45.95	714.06	3.95798	9.57E-06
14915	'Guca2a'	4642.95	1138.6	-2.02778	0.01122
14938	'Gzma'	78.44	7.85	-3.32106	0.01668
15186	'Hdc'	8.33	105.05	3.65647	0.01749
15396	'Hoxa11'	23.7	540.48	4.51144	1.13E-04
15404	'Hoxa7'	617.15	266.42	-1.21192	0.00367
15446	'Hpgd'	912.08	170.45	-2.41986	0.00810
15486	'Hsd17b2'	1014.57	352.52	-1.52508	0.00109
15957	'Ifit1'	144.24	40.99	-1.81493	0.04665
16010	'Igfbp4'	2857.47	8249.89	1.52964	0.01549
16147	'Ihh'	1486.72	503.44	-1.56225	0.00521
16181	'Il1rn'	9959.74	4353.47	-1.19394	0.03082
16193	'Il6'	3.37	152.15	5.49566	0.00378
16392	'Isl1'	3.01	172.16	5.83598	0.03044
16574	'Kif5c'	107.11	385.97	1.84943	0.02303
17002	'Ltf'	19.92	350.58	4.13712	0.02610
170761	'Pdzd3'	94.92	20.04	-2.24358	0.03279
171168	'Acer1'	357.54	51.13	-2.80573	2.97E-04
17119	'Mxd1'	4389.81	1873.66	-1.22830	0.04504
17161	'Maoa'	1637.21	461.21	-1.82774	4.41E-08
17287	'Mep1a'	736.77	143.03	-2.36493	1.12E-08
17288	'Mep1b'	809.45	301.67	-1.42396	0.01331
17301	'Foxd2'	920.17	177.27	-2.37593	1.26E-05
17387	'Mmp14'	1903.1	6420.49	1.75433	0.04467

17701	'Msx1'	342.64	35.76	-3.26022	6.45E-13
17751	'Mt3'	36.98	321.82	3.12132	2.65E-04
17829	'Muc1'	6.32	259.74	5.36002	6.71E-04
18602	'Padi4'	29.27	297.82	3.34704	0.01968
18858	'Pmp22'	7089.57	2673.73	-1.40685	5.64E-04
18946	'Pnliprp1'	94.79	582.78	2.62009	0.00202
19242	'Ptn'	123.43	396.12	1.68231	0.01128
19277	'Ptpro'	155.41	999.67	2.68540	0.01072
19279	'Ptprr'	528.34	148.24	-1.83353	0.02425
19752	'Rnase1'	217.72	759.6	1.80278	0.02305
20201	'S100a8'	44.41	482.03	3.44017	0.02556
20310	'Cxcl2'	42.81	574.06	3.74522	0.00570
20341	'Selenbp1'	1362.6	361.28	-1.91518	7.13E-08
20363	'Selenop'	2424.21	995.42	-1.28413	5.32E-04
20500	'Slc13a2'	558.86	104.55	-2.41834	8.22E-04
20510	'Slc1a1'	43.19	270.01	2.64430	2.83E-04
20532	'Slc3a1'	367.2	79.28	-2.21147	1.29E-05
208677	'Creb3l3'	278.8	100.12	-1.47753	0.03308
20868	'Stk10'	259.02	90.47	-1.51761	0.02163
209378	'Itih5'	331.67	78.07	-2.08683	0.00639
209601	'Erich3'	3.46	53.64	3.95388	0.01702
212190	'Ubxn10'	150.06	400.7	1.41697	0.00378
216225	'Slc5a8'	502.98	119.19	-2.07722	0.00290
21685	'Tef'	1045.87	471.13	-1.15051	0.00186
217316	'Slc16a5'	352.7	104.68	-1.75246	0.00205
217328	'Myo15b'	4263	1741.2	-1.29179	3.02E-05
218454	'Lhfpl2'	623.28	204.01	-1.61121	5.64E-04
21946	'Pglyrp1'	7003.77	14715.79	1.07116	1.33E-04
22337	'Vdr'	1256.76	269.98	-2.21878	2.82E-06
223706	'Cyp2d34'	472.02	130.23	-1.85785	2.69E-06
224796	'Clic5'	300.23	99.42	-1.59444	0.00169
225997	'Trpm6'	396.42	101.6	-1.96407	0.01902
226999	'Slc9a2'	2135.97	860.43	-1.31177	1.87E-05
230863	'Sh2d5'	81.98	373.41	2.18741	0.05000

231293	'Cwh43'	3928.8	1382.35	-1.50696	0.00240
231507	'Plac8'	33154.5	15671.28	-1.08108	0.00109
234311	'Ddx60'	109.78	27.92	-1.97505	0.03977
234671	'Ces2c'	807.97	164.82	-2.29341	1.26E-05
234673	'Ces2e'	1338.51	463.02	-1.53149	0.00193
235674	'Acaa1b'	133.14	11.86	-3.48913	0.02570
236539	'Phgdh'	701.92	1731.36	1.30251	0.04085
238055	'Apob'	623.33	177.36	-1.81330	0.00670
239273	'Abcc4'	720.29	1792.47	1.31529	2.18E-06
241275	'Noxa1'	561.17	1545.04	1.46115	0.01770
24131	'Ldb3'	18.65	335.47	4.16898	7.00E-09
245195	'Retnlg'	17.78	193.85	3.44684	0.03623
246221	'Mpst'	2749.33	1020.96	-1.42914	0.00544
26357	'Abcg2'	416.6	141.97	-1.55309	0.00178
268663	'Cdhr2'	4963.09	2186.8	-1.18242	5.50E-04
26918	'Ern2'	199.59	451.85	1.17878	0.02508
30943	'Prss30'	1171.36	288.32	-2.02245	2.86E-08
319848	'Slc17a4'	547.05	207.05	-1.40168	0.00109
331063	'Gsdmc2'	1242.9	474.95	-1.38787	0.02053
338521	'Fa2h'	2025.85	506.66	-1.99943	3.70E-08
381217	'Fam189a2'	1007.3	202.28	-2.31606	1.33E-04
394436	'Ugt1a1'	307.98	22.64	-3.76599	9.94E-06
406219	'Krt87'	29.23	229.37	2.97229	3.70E-04
435921	'Clec2f'	5.4	112.65	4.38380	1.83E-04
53315	'Sult1d1'	1193.63	212.45	-2.49016	0.00777
53945	'Slc40a1'	1209.2	517.22	-1.22520	0.00603
54123	'Irf7'	1519.02	399.74	-1.92599	2.00E-08
54159	'Rnase2b'	3.61	130.36	5.17632	4.70E-04
545902	'Ptprh'	1473.54	501.45	-1.55511	9.61E-05
54611	'Pde3a'	539.48	233.81	-1.20620	0.03201
54613	'St3gal6'	351.84	799.57	1.18428	0.03632
55925	'Syt8'	725.4	279.77	-1.37453	0.02761
56325	'Abcb9'	167.48	704.22	2.07203	0.00465
56362	'Sult1b1'	761.46	272.09	-1.48467	5.64E-04

56643	'Slc15a1'	94.97	19.52	-2.28281	0.04448
56742	'Psrc1'	138.7	632.09	2.18819	0.00112
57349	'Ppbp'	12.2	93.2	2.93330	0.04381
57738	'Slc15a2'	75.45	3.9	-4.27576	0.00112
58210	'Sectm1b'	1518.39	474.88	-1.67691	8.06E-11
60527	'Fads3'	469.77	974.73	1.05304	0.01702
620235	'Siglec15'	12.62	107	3.08352	0.04249
622434	'Arhgef26'	268.25	1036.06	1.94944	6.76E-04
624681	'Btnl6'	190.25	37.78	-2.33233	0.01702
632126	'Btnl4'	233.11	59.27	-1.97556	5.89E-04
64138	'Ctsz'	2527.11	5316.37	1.07295	0.00600
64898	'Lpin2'	1055.07	520.04	-1.02066	0.01661
66071	'Ethe1'	4422.44	1419.87	-1.63908	1.19E-05
66183	'Sptssb'	3088.66	717.28	-2.10637	1.68E-04
66206	'1110059E24Rik'	245.28	721.03	1.55566	9.70E-05
66269	'Tmed6'	64.64	410.48	2.66680	0.00101
66601	'Tmigd1'	1099.75	285.8	-1.94412	1.20E-04
666339	'Muc3'	20279.19	6296.56	-1.68736	7.52E-04
667034	'Pnp2'	3.02	612.03	7.66334	1.94E-23
66861	'Dnajc10'	1514.44	4101.42	1.43734	2.53E-04
67071	'Rps6ka6'	25.04	132.85	2.40727	0.03057
67088	'Cand2'	98.4	248.77	1.33806	0.04851
67315	'Ceacam12'	415.13	62.4	-2.73385	0.00131
67432	'Hoga1'	18.93	258.28	3.77046	0.04223
67569	'Mgat4c'	4093.94	1545.49	-1.40543	0.00127
67855	'Asprv1'	20.24	126.92	2.64847	0.00553
68778	'Gucd1'	1951.89	790.05	-1.30485	0.00110
69036	'Zg16'	9002.68	4228.29	-1.09028	0.02528
69824	'Glod5'	337.27	126.38	-1.41613	0.03203
69864	'1810065E05Rik'	7305.09	1043.15	-2.80796	1.20E-14
70045	'2610528A11Rik'	5339.68	996.41	-2.42194	5.96E-21
70163	'Lypd8'	40312.13	17640.89	-1.19229	0.00109
70551	'Tmtc4'	593.86	1557.39	1.39094	5.79E-06
71207	'Nudt4'	5425.38	2546.97	-1.09094	0.00544

71597	'Isx'	2240.73	838.81	-1.41755	0.04015
71664	'Mettl7b'	1812	307.38	-2.55947	2.50E-06
71853	'Pdia6'	3085.54	6574.31	1.09132	0.00869
71862	'Gpr160'	264.46	79.91	-1.72657	0.00160
72040	'Cdhr5'	7238	3295.08	-1.13528	0.01101
72074	'Anks4b'	725.33	327.51	-1.14709	0.00956
72082	'Cyp2c55'	3500.57	120.11	-4.86519	0.01879
72303	'Cyp2c65'	2326.22	265.89	-3.12909	8.46E-20
72393	'Faim2'	157.64	1865.06	3.56455	7.78E-06
72431	'Ceacam18'	84.58	9.84	-3.10319	0.04743
72709	'C1qtnf6'	422.28	1361.97	1.68943	0.00639
73833	'Fam98c'	1235.18	469.83	-1.39452	2.69E-06
74134	'Cyp2s1'	772.61	232.49	-1.73261	1.96E-05
74556	'Themis3'	399.74	130.11	-1.61935	0.01331
74591	'Abca12'	142.88	25.66	-2.47742	0.04217
74732	'Stx11'	154.65	411.18	1.41079	0.03089
74754	'Dhcr24'	17828.09	6983.41	-1.35215	0.01387
76282	'Gpt'	311.52	76.3	-2.02951	0.00967
76787	'Ppfia3'	572.28	226.71	-1.33590	5.18E-04
76872	'Ccdc116'	307.36	117.86	-1.38290	0.02128
77125	'Il33'	209.78	1073.62	2.35555	0.02305
77521	'Mtus2'	45.96	2.94	-3.96592	0.02167
77996	'Cutal'	940.4	452.81	-1.05436	0.01724
81840	'Sorcs2'	529.19	1199.67	1.18079	0.01867
93838	'Dqx1'	490.1	199.21	-1.29880	0.03448
94071	'Clec2h'	1830.12	472.81	-1.95260	0.01928
98396	'Slc41a1'	661.99	1557.33	1.23419	0.00701
99663	'Clca4a'	10306.22	3589.66	-1.52160	4.88E-04

Supplementary Table. S3: List of DEGs Between APC Mut Tumors and APC;KRAS Mut Tumors

Gene ID	Gene Symbol	*APC;KRAS* with DSS Average Read Count	*APC* with DSS Average Read Count	log2	Qvalue
100038882	'Isg15'	63.06	267.16	2.08286	0.03379
100041546	'Ly6c2'	21.63	151.2	2.80518	0.00706
100041952	'Defa34'	1.15	68.79	5.89975	8.91E-06
100043899	'R3hdml'	1811.32	723.65	-1.32369	0.02590
100294660	'Defa2'	74.83	1035.65	3.79074	0.02494
100504232	'Mptx2'	1.16	271.92	7.87505	9.64E-05
100689	'Spon2'	216.33	485.27	1.16557	0.00824
100702	'Gbp6'	27.05	244.54	3.17644	9.99E-05
100862126	'Defa36'	38.82	622.84	4.00397	2.28E-08
103142	'Rdh9'	405.24	1897.4	2.22716	0.02031
104001	'Rtn1'	9.3	46.25	2.31365	0.01387
107605	'Rdh1'	121.47	548.28	2.17429	0.02805
108013	'Celf4'	73.01	16.37	-2.15712	0.00188
108723	'Card11'	69.29	199.72	1.52723	8.44E-04
109050	'Inka2'	250.12	44.7	-2.48423	9.86E-08
109820	'Pgc'	61.67	12.14	-2.34500	0.03004
110895	'Slc9a4'	267.79	121.58	-1.13922	0.00985
114301	'Palmd'	362.97	123.55	-1.55474	4.21E-05
11459	'Acta1'	33.6	96.58	1.52348	0.00161
11516	'Adcyap1'	55.36	8	-2.79061	1.40E-06
11534	'Adk'	1790.87	797.51	-1.16708	0.00271
116847	'Prelp'	185.51	470.11	1.34147	1.77E-08
117591	'Slc2a9'	195.47	92.5	-1.07948	0.03381
11784	'Apba2'	3.77	41.01	3.44251	0.04490
118449	'Synpo2'	39.21	295.86	2.91544	0.01513
118453	'Mmp28'	613.65	246.05	-1.31844	0.02882
118567621	'LOC118567621'	208.01	695.81	1.74205	0.04903
118567918	'LOC118567918'	321.98	1198.34	1.89599	0.02868
11921	'Atoh1'	244.66	109.28	-1.16282	0.01444

12124	'Bik'	132.68	265.5	1.00081	0.03269
12164	'Bmp8b'	218.33	66.66	-1.71161	0.01513
12227	'Btg2'	4407.23	1927.55	-1.19311	1.12E-05
12268	'C4b'	188.64	802.49	2.08887	0.03639
12322	'Camk2a'	28.71	95.96	1.74084	0.01235
12332	'Capg'	3967.73	1900.58	-1.06187	0.02497
12443	'Ccnd1'	7320.92	3156.84	-1.21354	1.77E-08
12444	'Ccnd2'	16439.66	3629.58	-2.17931	1.17E-06
12450	'Ccng1'	4053.59	1277.78	-1.66557	7.13E-19
12479	'Cd1d1'	117.04	308.45	1.39804	0.02496
12505	'Cd44'	8262.88	3785.81	-1.12604	2.99E-04
12514	'Cd68'	97.9	273.13	1.48014	0.04664
12516	'Cd7'	4.85	26.51	2.44953	0.01333
12518	'Cd79a'	24.59	167.02	2.76370	0.00698
12552	'Cdh11'	113.08	351.83	1.63753	0.04884
12578	'Cdkn2a'	1457.38	386.18	-1.91603	0.00125
12638	'Cftr'	1459.1	483.62	-1.59314	1.29E-04
12715	'Ckm'	0.42	19.82	5.57554	0.04567
12759	'Clu'	20524.7	8526.74	-1.26730	0.02031
12797	'Cnn1'	42.16	1386.51	5.03938	0.03902
12977	'Csf1'	254.05	611.51	1.26730	0.03595
13036	'Ctsh'	3209.13	1494.89	-1.10215	1.40E-06
13070	'Cyp11a1'	295.72	97.43	-1.60179	0.03141
13239	'Defa5'	35.11	799.29	4.50873	0.00271
13363	'Dhh'	40.61	6.16	-2.71959	0.00788
13386	'Dlk1'	10.94	172.44	3.97873	9.22E-06
13507	'Dsc3'	1.52	52.3	5.10618	0.02240
13512	'Dsg3'	213.53	21.93	-3.28348	6.67E-14
13522	'Adam28'	82.2	2.52	-5.02691	9.53E-16
13601	'Ecm1'	2852.57	1311.24	-1.12133	5.36E-04
13612	'Edil3'	5.57	31.43	2.49758	0.02337
13638	'Efna3'	244.87	71.81	-1.76977	5.53E-05
13717	'Eln'	119.53	611.42	2.35483	0.02732
13730	'Emp1'	7153.12	2534.3	-1.49699	0.02240

13849	'Ephx1'	1871.07	575.5	-1.70099	5.36E-04
13884	'Ces1c'	17.39	64.75	1.89635	0.03929
14067	'F5'	248.38	63.07	-1.97748	0.00322
14114	'Fbln1'	537.2	1458.8	1.44126	1.14E-04
14133	'Fcna'	168.99	53.53	-1.65861	0.00583
14168	'Fgf13'	76.48	11.74	-2.70387	1.05E-06
14183	'Fgfr2'	448.39	1391.08	1.63338	2.66E-04
14570	'Arhgdig'	255.47	80.93	-1.65845	2.28E-08
14579	'Gem'	73.81	203.19	1.46092	0.04600
14584	'Gfpt2'	13.9	46.44	1.74027	0.03141
14594	'Ggta1'	291.8	125.91	-1.21262	7.47E-04
14758	'Gpm6b'	114.33	319.44	1.48231	0.03162
14860	'Gsta4'	4357.75	1036.34	-2.07208	3.79E-11
14873	'Gsto1'	7947.48	3852.57	-1.04468	0.03149
15015	'H2-Q4'	506.16	1510.91	1.57775	0.01610
15114	'Hap1'	93.24	198.8	1.09225	0.04862
15958	'Ifit2'	87.57	620.11	2.82395	2.58E-05
15985	'Cd79b'	6	57.94	3.27265	0.01088
16069	'Jchain'	2490.7	21038.44	3.07840	0.04613
16425	'Itih2'	39.49	7.31	-2.43356	0.03091
16450	'Jag2'	720.99	332.94	-1.11471	0.00148
16511	'Kcnh2'	67.03	160.57	1.26035	0.01955
16574	'Kif5c'	354.25	85.82	-2.04532	4.63E-04
16663	'Krt13'	338.3	112.2	-1.59225	9.30E-04
16667	'Krt17'	84.97	733.73	3.11029	1.65E-05
16792	'Laptm5'	331.71	1043.17	1.65299	0.02031
16918	'Mycl'	703.02	1660.34	1.23983	5.35E-04
170638	'Hpcal4'	80.1	26.43	-1.59948	0.01947
170771	'Khdrbs2'	118.73	258.44	1.12215	0.00521
17105	'Lyz2'	1164.28	5054.13	2.11803	0.01600
17110	'Lyz1'	10457.54	31478.66	1.58983	4.70E-11
171207	'Arhgap4'	34.85	99.87	1.51898	0.02286
17153	'Mal'	786.11	209.39	-1.90855	0.00439
17174	'Masp1'	19.34	88.99	2.20193	0.00270

17228	'Cma1'	384.87	65.79	-2.54834	1.70E-04
17314	'Mgmt'	149.58	32.14	-2.21857	1.76E-04
17385	'Mmp11'	80.39	204.07	1.34402	0.03862
17755	'Map1b'	74.11	166.63	1.16885	0.01173
17829	'Muc1'	479.52	1199.26	1.32247	0.03665
17869	'Myc'	2621.11	843.24	-1.63617	4.74E-04
18035	'Nfkbia'	1263.51	2567.84	1.02312	0.04057
18197	'Nsg2'	6.33	31.17	2.29929	0.04393
18553	'Pcsk6'	1810.19	688.64	-1.39431	0.00195
18612	'Etv4'	1641.59	504.93	-1.70095	4.03E-08
18645	'Pfn2'	63.36	274.01	2.11267	0.00349
18712	'Pim1'	568.35	1352.14	1.25038	0.03036
18751	'Prkcb'	24.75	90.11	1.86403	0.03736
18802	'Plcd4'	22.99	0.63	-5.18485	8.02E-04
19128	'Pros1'	997.51	439.47	-1.18258	0.01185
19142	'Prss12'	649.02	187.97	-1.78774	4.85E-05
192198	'Lrrc4'	22.54	77.07	1.77377	0.01642
19245	'Ptp4a3'	2056.76	663.8	-1.63156	3.07E-06
19270	'Ptprg'	1842.32	811.57	-1.18273	0.02271
19281	'Ptprt'	50.23	157.31	1.64705	8.02E-04
192897	'Itgb4'	8238.37	4087.81	-1.01103	0.00949
19415	'Rasal1'	151.71	54.68	-1.47235	0.02973
194655	'Klf11'	258.82	603.82	1.22218	0.00101
19659	'Rbp1'	1370.86	659.9	-1.05476	0.00196
19703	'Renbp'	57.99	138.43	1.25528	0.02088
20531	'Slc34a2'	77.41	252.83	1.70749	0.00111
20671	'Sox17'	100.54	519.4	2.36904	0.01711
20713	'Serpini1'	11.94	85.72	2.84375	0.00281
20725	'Serpinb8'	78.61	19.08	-2.04301	0.00288
20750	'Spp1'	8427.5	1912.2	-2.13987	1.58E-04
209588	'Sectm1a'	74.32	348.96	2.23128	0.01440
211389	'Suox'	342.93	142.99	-1.26200	0.01564
212439	'AA986860'	1246.09	447.37	-1.47787	4.25E-13
213391	'Rassf4'	42.79	178.83	2.06338	1.40E-06

213409	'Lemd1'	75.52	23.06	-1.71170	0.00106
215798	'Adgrg6'	222.46	107.16	-1.05380	0.03379
216867	'Slc16a11'	152.14	70.81	-1.10330	0.00635
216892	'Spns2'	2168.49	740.94	-1.54927	6.03E-11
217169	'Tns4'	6557.89	1296.81	-2.33826	2.30E-08
217212	'Pyy'	1268.55	3903.11	1.62144	0.02380
217653	'Mis18bp1'	95.42	197.87	1.05220	0.00325
217830	'Dglucy'	850.09	313.52	-1.43905	5.57E-08
21814	'Tgfbr3'	17.44	119.49	2.77606	0.03139
218772	'Rarb'	32.33	233.59	2.85324	0.00583
218820	'Zfp503'	197.95	663.88	1.74579	0.04583
21909	'Tlx2'	0.21	37.45	7.45699	0.00147
21956	'Tnnt2'	396.23	132.89	-1.57614	5.90E-12
21987	'Tpd52l1'	256.3	57.02	-2.16833	2.29E-05
22035	'Tnfsf10'	120.03	595.8	2.31142	2.03E-04
22164	'Tnfsf4'	19.91	2.33	-3.09342	0.03149
22169	'Cmpk2'	86.91	217.44	1.32299	0.00114
22329	'Vcam1'	87.02	318.57	1.87217	0.03562
223672	'Apol9a'	14.37	68.11	2.24435	0.02548
22401	'Zmat3'	1628.53	450.66	-1.85347	1.52E-09
224090	'Tmem44'	38.02	148.78	1.96850	0.00211
22415	'Wnt3'	15.86	95.67	2.59263	8.38E-05
224617	'Tbc1d24'	145.83	61.44	-1.24710	0.00661
225608	'Sh3tc2'	303.9	73.87	-2.04060	1.06E-04
225870	'Rin1'	253.65	95.34	-1.41163	4.14E-04
226041	'Pgm5'	30.71	347.54	3.50046	9.94E-04
226101	'Myof'	2832.79	1159.11	-1.28920	1.41E-06
226922	'Kcnq5'	32.48	6.38	-2.34855	0.00243
228413	'Prrg4'	370.75	118.48	-1.64580	8.02E-05
228432	'Ano3'	101.84	1.47	-6.11845	4.38E-15
229302	'Tm4sf4'	27.44	151.05	2.46071	0.00762
230784	'Sesn2'	288.69	96.64	-1.57887	0.00161
230903	'Fbxo44'	59.27	129.35	1.12600	0.02795
230991	'Fndc10'	555.76	186.58	-1.57471	2.51E-04

231296	'Lrrc66'	1204.78	404.96	-1.57293	0.00121
231382	'Tmprss11d'	68.63	1.88	-5.18726	2.40E-09
232370	'Clstn3'	3.76	67.14	4.15843	0.02660
232813	'Shisa7'	20.63	75.39	1.86949	0.02034
233066	'Syne4'	33.05	129.02	1.96496	0.00645
233733	'Galnt18'	38.2	120.86	1.66175	0.03665
234593	'Ndrg4'	28.96	78.55	1.43940	0.02575
236573	'Gbp9'	59.75	358.44	2.58469	5.14E-04
236643	'Sytl5'	35.41	11.99	-1.56220	0.04150
237436	'Gas2l3'	82.47	169.34	1.03809	0.00920
237523	'Ptprq'	33.56	4.08	-3.04163	0.01513
237759	'Col23a1'	28.04	203.34	2.85847	0.01615
237847	'Rtn4rl1'	172.82	568.67	1.71828	6.02E-04
238266	'Syt16'	191.4	52.86	-1.85622	3.07E-07
23853	'Def6'	330.22	93.05	-1.82743	0.01513
239673	'Krt90'	1322.01	241.4	-2.45321	0.00799
240754	'Lax1'	10.61	103.54	3.28606	0.02318
24110	'Usp18'	47.21	262.92	2.47743	0.00347
241950	'Bbs12'	135.4	60.15	-1.17053	0.01513
242523	'Dmrta1'	44.97	0.84	-5.74893	1.40E-06
242721	'Klhdc7a'	343.3	91.9	-1.90132	0.00196
242819	'Rundc3b'	176.9	73.23	-1.27242	0.00446
243529	'H1f10'	99.48	318.49	1.67870	0.04936
244202	'Nlrp10'	341.09	119.5	-1.51313	0.03260
244579	'Tox3'	229.41	502.6	1.13146	0.00371
245527	'Eda2r'	493.38	72.83	-2.76014	1.52E-09
246086	'Onecut3'	54.48	2.57	-4.40501	6.28E-06
258571	'Olfr1033'	1399.2	113.21	-3.62752	4.32E-11
26359	'Anxa10'	32.28	0.25	-6.99583	7.33E-06
26366	'Ceacam10'	615.27	1582.55	1.36296	0.04839
26458	'Slc27a2'	16.34	71.77	2.13493	0.00462
26570	'Slc7a11'	1449.57	663.22	-1.12806	0.01280
268291	'Rnf217'	271.6	123.27	-1.13968	0.00397
268527	'Greb1'	204.8	35.53	-2.52715	4.24E-05

26875	'Pclo'	26.41	3.66	-2.85168	4.75E-04
268780	'Egflam'	175.22	394.25	1.16995	0.00303
269152	'Kif26b'	173.52	626.97	1.85326	0.04755
269356	'Slc4a11'	1039.34	190.04	-2.45130	5.18E-25
269637	'Cnpy1'	5.58	38.22	2.77604	0.00188
26968	'Islr'	38.05	160.15	2.07344	0.02738
27015	'Polk'	234.38	101.76	-1.20372	2.10E-05
271375	'Cd200r2'	207.92	96.31	-1.11035	0.00247
27226	'Pla2g7'	1507.72	567.84	-1.40882	0.00950
27280	'Phlda3'	1991.35	573.09	-1.79691	5.59E-11
27355	'Pald1'	159.49	354.06	1.15052	0.01332
27409	'Abcg5'	87.72	263.19	1.58520	0.01541
279653	'Pcdh19'	21.51	297.2	3.78812	2.98E-09
29818	'Hspb7'	2.7	77.89	4.85209	0.01513
29858	'Pmm1'	1439.94	647.4	-1.15329	4.12E-09
320159	'Togaram2'	5.61	31.68	2.49696	0.04800
320508	'Cachd1'	312.81	722.08	1.20686	0.03041
320679	'Samd12'	510.77	178.04	-1.52046	5.18E-06
320712	'Abi3bp'	18.84	116.31	2.62631	0.01600
320825	'Samd5'	623.53	131.62	-2.24408	3.18E-09
328258	'Slc25a48'	67.15	11.85	-2.50195	0.03385
330450	'Far2'	12.47	44.7	1.84203	0.02494
330812	'Rnf150'	117.42	293.51	1.32170	0.04884
380660	'Acss3'	231.52	31.54	-2.87573	1.83E-05
381269	'Mreg'	17.59	121.49	2.78827	8.91E-05
381290	'Atp2b4'	80.33	215.1	1.42098	0.01895
382059	'Defa22'	21.8	347.8	3.99606	0.01996
435684	'Shf'	1684.23	651.5	-1.37026	1.04E-04
51791	'Rgs14'	28.48	98.12	1.78446	3.65E-04
53321	'Cntnap1'	19.96	54.01	1.43584	0.03896
545260	'Arsi'	62.43	245.62	1.97620	0.02398
545481	'Arhgap40'	92.54	25.66	-1.85076	7.63E-04
554292	'Methig1'	17.52	138.1	2.97822	1.08E-04
55985	'Cxcl13'	39.3	382.07	3.28120	1.40E-06

55987	'Cpxm2'	58.94	173.28	1.55581	0.02798
56047	'Msln'	1487.04	301.62	-2.30163	5.70E-07
56078	'Car5b'	106.98	36.6	-1.54752	0.03752
56325	'Abcb9'	549.42	227.53	-1.27183	0.00532
56742	'Psrc1'	752.54	190.67	-1.98066	1.09E-04
58210	'Sectm1b'	829.16	3976.19	2.26166	3.96E-05
58226	'Cacna1h'	31.65	291.79	3.20472	0.00180
58238	'Fam181b'	0.42	23.36	5.81260	0.03802
58805	'Mlxipl'	62.03	187.12	1.59297	0.03869
59020	'Pdzk1'	5.7	58.21	3.35152	0.00149
59095	'Fxyd6'	1102.67	4398.06	1.99586	1.59E-04
64929	'Scel'	351.77	93.08	-1.91805	6.88E-05
66214	'Rgcc'	1173.77	239.67	-2.29205	1.52E-09
66283	'Gkn1'	78.64	4.08	-4.26800	2.70E-11
66438	'Hamp2'	3.81	60.66	3.99253	7.33E-06
666794	'Rbm24'	171.76	416.43	1.27764	0.04936
66993	'Smarcd3'	47.47	125.97	1.40809	0.00698
67133	'Gp2'	0.21	37.78	7.46729	6.34E-05
67169	'Nradd'	100.91	241.65	1.25989	0.01512
67470	'Abcg8'	39.29	139.5	1.82787	0.02417
67775	'Rtp4'	108.04	271.56	1.32966	0.01955
67859	'Cysrt1'	56.62	9.87	-2.51961	8.91E-05
68009	'Defa20'	56.33	916.01	4.02335	0.01620
68169	'Ndnf'	311.41	83.71	-1.89540	0.01595
68311	'Lypd2'	0.21	9.81	5.54286	0.03686
68337	'Crip2'	2491.93	924.72	-1.43017	0.00371
68617	'Mtcl1'	526.15	246.02	-1.09670	0.04050
68713	'Ifitm1'	676.12	2504.21	1.88900	0.02034
68794	'Flnc'	69.04	353.1	2.35455	0.02691
68888	'Gkn3'	143.28	33.92	-2.07852	0.04969
68918	'1190005I06Rik'	134.44	43.84	-1.61673	0.04598
69065	'Chac1'	27.47	88.36	1.68568	0.00698
69121	'Chrdl2'	4.59	61.06	3.73517	0.00698

69327	'1700007K13Rik'	19.42	2.4	-3.01602	0.00446
69534	'Avpi1'	1156.48	530.23	-1.12504	1.21E-06
69656	'Pir'	451.44	210.76	-1.09895	0.00896
69675	'Pxdn'	537.77	1367.81	1.34681	0.02294
69816	'Mzb1'	46.31	370.48	2.99999	0.01185
70377	'Derl3'	17.16	96.92	2.49743	0.03004
70435	'Inf2'	1456.51	685.46	-1.08737	9.54E-05
70785	'Dennd1c'	45.63	137.72	1.59380	0.01693
70887	'Dmrtc1a'	0	32.87	20.47950	8.91E-06
70956	'Tex19.2'	0.41	15.6	5.23757	0.04570
71146	'Golga7b'	3.05	30.93	3.34220	0.00371
71306	'Mfap3l'	202.17	84.82	-1.25305	0.01128
71884	'Chit1'	601.61	69.63	-3.11113	2.17E-09
71897	'Lypd6b'	65.94	155.72	1.23979	0.04960
72043	'Sulf2'	4545.75	2028.48	-1.16412	0.01743
73284	'Ddit4l'	321.31	71.07	-2.17658	6.18E-06
73338	'Itpripl1'	753.66	345.8	-1.12399	1.34E-04
73649	'Cybrd1'	179.12	31.27	-2.51787	5.59E-11
74096	'Hvcn1'	31.67	106.49	1.74934	0.01385
74182	'Gpcpd1'	880.29	1890.05	1.10238	0.03736
74490	'Mamstr'	12.84	53.43	2.05729	0.03409
74645	'Tent5c'	177.06	494.09	1.48057	0.02590
76130	'Las1l'	1289.18	642.25	-1.00524	0.00100
76267	'Fads1'	624.28	1937.33	1.63380	4.63E-04
76477	'Pcolce2'	71.28	1.93	-5.20690	6.90E-08
77125	'Il33'	1879.19	496.62	-1.91989	9.94E-04
78317	'Ccdc88b'	101.24	410.79	2.02059	2.48E-04
78405	'Ntf5'	56.71	19.46	-1.54308	0.04533
78789	'Vsig1'	1272.1	591.11	-1.10573	0.02647
79201	'Tnfrsf23'	930.87	369.96	-1.33122	2.95E-07
79202	'Tnfrsf22'	283.79	112.75	-1.33166	0.02240
80880	'Kank3'	196.66	55.28	-1.83081	1.00E-06
83397	'Akap12'	44.72	117.31	1.39132	0.00847

93695	'Gpnmb'	23.38	290.55	3.63552	0.00263
93732	'Acox2'	297.43	64.7	-2.20076	4.85E-05
93735	'Wnt16'	10.4	97.77	3.23232	0.00325
94180	'Acsbg1'	620.65	218.86	-1.50374	0.00742
99899	'Ifi44'	10.43	64.6	2.63119	0.00401
